# The gut-heart axis in heart failure: from bidirectional pathophysiological mechanisms to integrative therapeutic strategies

**DOI:** 10.3389/fmicb.2026.1831648

**Published:** 2026-05-11

**Authors:** Xue Rui, Lihong Wu, Hang Ren, Shanshan Jia, Jinjin Dou, Xiwu Zhang

**Affiliations:** 1 Graduate School, Heilongjiang University of Chinese Medicine, Harbin, China; 2 Experimental Teaching and Practical Training Center, Heilongjiang University of Chinese Medicine, Harbin, China; 3 The Fourth Affiliated Hospital of Harbin Medical University, Harbin Medical University, Harbin, China; 4 The Fourth Affiliated Hospital of Heilongjiang University of Chinese Medicine, Heilongjiang University of Chinese Medicine, Harbin, China

**Keywords:** gut microbiota dysbiosis, gut-heart axis, heart failure, integrative medicine, traditional Chinese medicine

## Abstract

Heart failure (HF), as the terminal stage of cardiovascular diseases, involves complex pathophysiological mechanisms that extend beyond the heart itself and involve the interactive regulation of multiple organ systems. The gut-heart axis theory reveals that the gut microbiota, functioning as a micro-organ, plays a core role in the development and progression of HF through bidirectional regulatory mechanisms. HF-induced hemodynamic disturbances reshape the gut microenvironment, leading to the collapse of intestinal physical and immune barrier functions. In this altered microenvironment, dysregulated gut bacteria release excessive pro-inflammatory metabolites while reducing protective signaling molecules, thereby activating inflammatory pathways including Toll-Like Receptor 4/Nuclear Factor-κB and NOD-like receptor protein 3 inflammasomes. Traditional Chinese medicine (TCM) is the most import integrative medicine to treat HF, and the combination of TCM and western medicine have reached certain progress. This article aims to describe the pathological feedback mechanism between HF and gut microbiota, as long as summary how TCM and western medicine treat HF through regulate gut-heart axis. In specific, modern medicine optimizes hemodynamics to improve the macroscopic microenvironment, thereby establishing a foundation for gut microbiota remodeling. TCM exerts holistic regulatory advantages through multiple components and targets, inhibiting the production of gut-derived inflammatory factors at their source by reshaping the oxygen gradient, providing prebiotic substrates, and repairing intestinal barrier integrity. These could provide a theoretical basis and research reference for precision-oriented HF prevention and management from the perspective of intestinal microecology.

## 1 Introduction

As the final stage of various cardiovascular diseases, HF remains a core challenge in the global public health field due to its high disease burden and severe prognosis (Wang Z. et al., 2023). According to statistics, the number of HF patients worldwide has exceeded 26 million and is showing a continuous upward trend (Hao et al., 2019; Groenewegen et al., 2020). In China, the HF prevalence rate has increased by 44% in the past 15 years, with the number of patients exceeding 13 million, more than 3.5 million new cases annually, and a 5-year mortality rate as high as 75.4% (Wang Z. et al., 2023; Pagnesi et al., 2022; Wang et al., 2021). In terms of medical economic burden, HF-related expenditures account for more than 30% of the total global cardiovascular disease expenditures, highlighting the urgency of HF prevention and control (Chioncel et al., 2024).

In recent years, the gut microbiota, as a key microbial organ in the human body, has been increasingly recognized for its core regulatory role in the gut-heart axis mechanism. HF-induced hemodynamic disturbances can cause mesenteric ischemia, hypoxia, and intestinal barrier damage, leading to the remodeling of the gut microbiota. This is characterized by reduced microbial diversity, an imbalance in the ratio of Bacillota/Bacteroidota, and an abnormal enrichment of opportunistic pathogens (Everard and Cani, 2014; Zhao S. et al., 2025; Zhang et al., 2025b). Simultaneously, the synergistic effect of gut microbiota metabolites, including trimethylamine N-oxide (TMAO) and short-chain fatty acids (SCFAs), and inflammatory pathways can exacerbate myocardial remodeling and functional decline, forming a vicious cycle of bidirectional feedback between HF and gut microbiota dysbiosis (Shi et al., 2023; Zhang et al., 2025a). Clinical studies have confirmed that intervention targeting beneficial bacteria such as *Akkermansia muciniphila* can improve HF-related metabolic disorders and reduce myocardial damage caused by gut microbiota-derived toxins, providing a novel target for HF prevention and treatment (Zhang et al., 2025b; Zhang L. et al., 2025).

While current conventional modern medical treatments have made significant progress in improving hemodynamics and blocking excessive neurohormonal activation (Rossignol et al., 2019; Bots et al., 2019). Its limitation lies in neglecting gut microbiota-mediated systemic inflammation and metabolic disorders. Especially in heart failure with preserved ejection fraction (HFpEF), conventional drugs have limited response, suggesting that targeting the gut microbiota may be a new therapeutic breakthrough. Furthermore, long-term use of diuretics and other drugs may lead to electrolyte imbalance, further exacerbating the deterioration of the gut microenvironment and dysbiosis, forming a negative feedback loop between treatment and pathology (Pagnesi et al., 2023). In terms of treatment strategies, in addition to the targeted gut microbiota therapy of modern medicine, TCM also shows unique overall regulatory advantages in regulating microbiota homeostasis, repairing the intestinal barrier and inhibiting fibrosis through multi-component and multi-target methods (Pagnesi et al., 2023; Roessler et al., 2025). Clinical evidence shows that the combination of traditional Chinese and Western medicine in the treatment of HF has a positive additive effect (Lin et al., 2014; Meng et al., 2018; Zhang et al., 2024a).

This review conducted a comprehensive literature search in PubMed, Google Scholar, CNKI, Wanfang, and VIP databases for publications from January 2015 to December 2025. For the original article, the searching timeline extend to January 2010–December 2025. The search strategy combined the following terms using Boolean operators (AND/OR): (“heart failure” OR “cardiac failure” OR “chronic heart failure”) AND (“gut microbiota” OR “intestinal microbiota” OR “intestinal flora” OR “gut microbiome”) AND (“traditional Chinese medicine” OR “Chinese patent medicine” OR “Chinese herbal medicine” OR “integrated Chinese and Western medicine”). Original research articles, systematic reviews, and meta-analyses published in English or Chinese were included. Conference abstracts, editorials, and studies unrelated to the gut-heart axis in HF were excluded.

Based on this, this review constructs a framework for the association between Western medicine subtypes/gradings and gut microbiota characteristics. We elucidate the correspondence between different clinical phenotypes and microbiome profiles by deeply analyzing the bidirectional interaction mechanism between HF and gut microbiota, and discuss the synergistic and toxicity-reducing pathways of regulating gut microbiota under the integrated traditional Chinese and Western medicine treatment strategy. This study aims to provide new biological insights and clinical references for the precise and multidimensional prevention and treatment of HF in the context of the gut-heart axis mechanism.

## 2 Bidirectional interaction mechanisms between HF and gut microbiota

### 2.1 HF-driven gut ecological reconstruction

#### 2.1.1 Hemodynamic abnormalities

**Figure 1:**
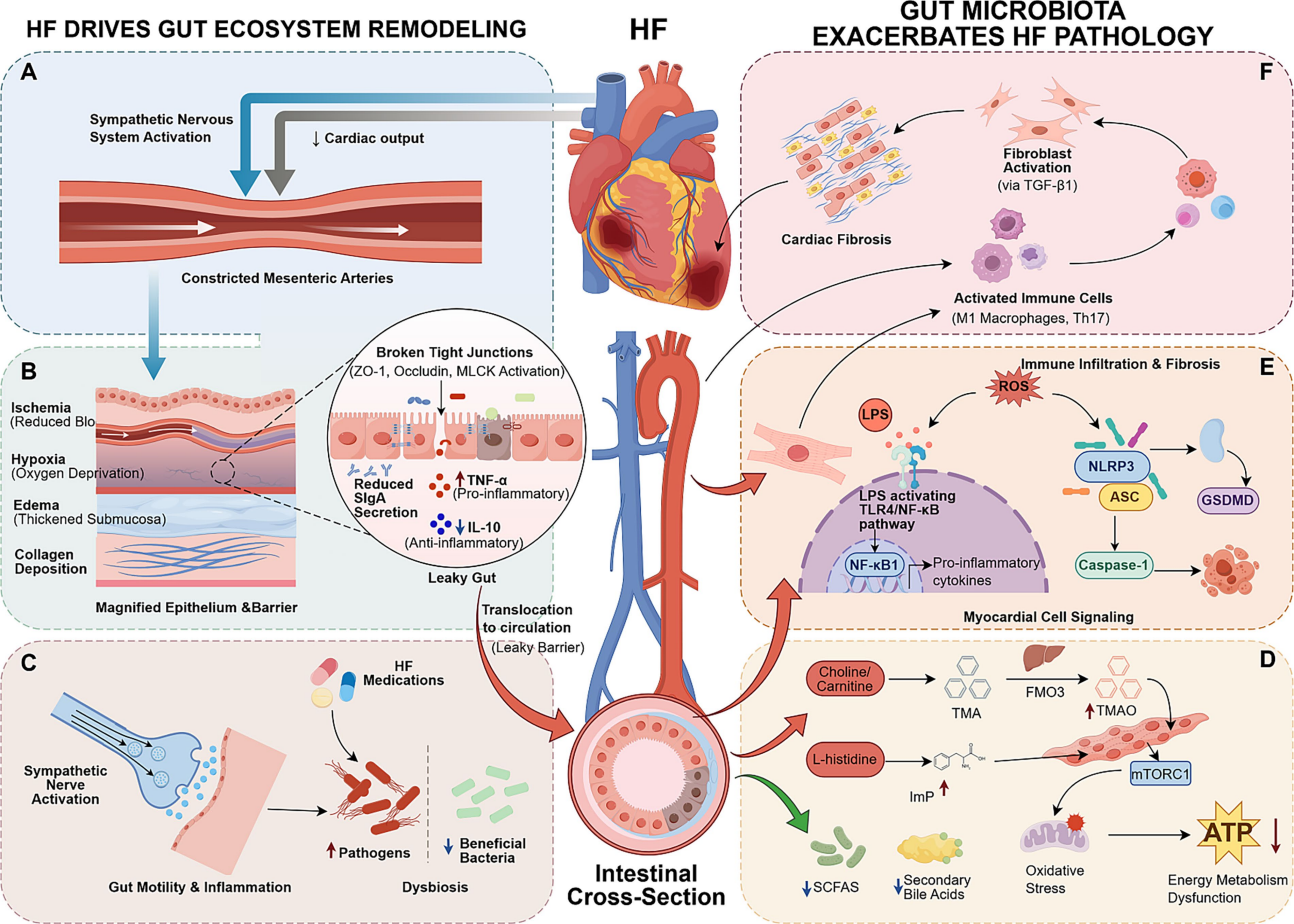
Bidirectional interaction mechanisms between HF and gut microbiota. Left panel: HF-driven gut ecological remodeling **(A–C)**. **(A)** Hemodynamic abnormalities lead to intestinal ischemia and hypoxia, disrupting the microenvironment for microbiota colonization. **(B)** Impaired intestinal barrier function induces microbiota translocation and immune imbalance. **(C)** Indirect synergistic effects of neuroendocrine activation and pharmacological interventions. Right panel: Pathological mechanisms of gut microbiota in reverse exacerbation of HF **(D–F)**. **(D)** Imbalance of microbiota-derived metabolites (pathology-promoting and protective molecules). **(E)** Microbiota-mediated activation of inflammatory pathways. **(F)** Synergistic damage via the gut-immune-heart axis (by figdraw).

Under HF conditions, a significant reduction in cardiac output triggers overactivation of the sympathetic nervous system and the renin-angiotensin-aldosterone system, leading to adaptive redistribution of systemic circulation (Hartupee and Mann, 2017). The key to this hemodynamic change lies in the inadequate intestinal perfusion and venous congestion caused by decreased cardiac output. Clinical observational studies have reported that systolic blood flow in the superior and inferior mesenteric arteries and celiac trunk of patients with HF is reduced by 30–43% compared to healthy individuals. The studies have further demonstrated that this leads to pathological aggravation of intestinal wall thickening due to edema and collagen deposition, with mucosal collagen content increasing 2.1 times compared to non-HF individuals. Furthermore, the degree of deposition is positively correlated with New York Heart Association (NYHA) classification (Sandek et al., 2014; Ikeda et al., 2017; Hao et al., 2024). This hemodynamic abnormality and structural remodeling, on the one hand, physically compresses the space for microbiota colonization, and on the other hand, destroys the integrity of the intestinal barrier, leading to an imbalance in the expression of tight junction proteins, thinning of the mucus layer, and the formation of a disordered microenvironment for interaction between microbiota and intestinal epithelial cells (Huo et al., 2021). Simultaneously, the retrograde circulation characteristics of intestinal villi make them susceptible to ischemia. HF results in a significant decrease in oxygen partial pressure at the villus tips compared to normal levels, creating an severely hypoxic microenvironment (Jodal and Lundgren, 1986). This further deteriorates the survival conditions of the gut microbiota. Under this pathological background, the proliferation of SCFA-producing bacteria, represented by *Lactobacillus*, is significantly inhibited. A clinical observational study reported that the abundance of SCFA-producing bacteria was reduced by approximately 45% compared to healthy individuals. This leads to insufficient SCFA production in the gut, weakening their regulatory role in intestinal epithelial cell repair and tight junction stability (Luedde et al., 2017). Conversely, hypoxia-tolerant pro-inflammatory bacteria exhibit selective enrichment, with *Prevotella* abundance increasing 2.3 times (Luedde et al., 2017). The abundance of *Desulfovibrio* increased threefold and was positively correlated with intestinal wall permeability. Based on animal and *in vitro* mechanistic studies, this type of bacteria may activate the intestinal mucosal Nuclear Factor-κB (NF-κB) pathway by releasing pro-inflammatory metabolites such as lipopolysaccharide (LPS) and hydrogen sulfide (H₂S), exacerbating barrier damage (Cui H. et al., 2023). Proteobacteria, as facultative anaerobic pathogens, also gain a competitive advantage and proliferate excessively (clinical observation) (Luedde et al., 2017), whose metabolites can enter the blood circulation through the damaged intestinal mucosa, amplifying the systemic inflammatory response (Chen et al., 2019). Therefore, the hemodynamic abnormalities caused by HF directly lead to the destruction of the physical structure of the intestinal microenvironment and oxygen supply imbalance. This deteriorated microenvironment is the initiating factor for subsequent loss of intestinal barrier function and severe dysbiosis (Figure 1A).

#### 2.1.2 Impaired intestinal barrier function

Under the direct effects of intestinal ischemia, hypoxia, and edema, the intestinal physical and immune barrier defense systems are disrupted, causing microbiota translocation and further exacerbating microbiota imbalance (Figure 1B). Tight junctions in the intestinal mucosa are a core structure preventing microbial translocation. Animal studies have shown that ischemia and hypoxia activate myosin light chain kinase (MLCK), leading to decreased levels of zonula occludens-1 (ZO-1) and occludin proteins, markedly increased intercellular gaps, and ultimately a significant increase in intestinal permeability (Al-Sadi et al., 2013). Clinical evidence further confirms that serum D-lactate levels, a marker of intestinal barrier damage, are 2.1 times higher in HF patients than in healthy individuals, and are positively correlated with plasma LPS levels, suggesting that damage to the intestinal physical barrier has led to abnormal entry of gut microbiota metabolites into the bloodstream (Li et al., 2020). The gut is the organ with the highest concentration of immune cells in the human body. Its intestinal mucosal immune barrier covers a total area of 400–600 square meters, serving as a key line of defense against bacterial invasion (Takiishi et al., 2017). As reviewed in the literature, in HF, intestinal ischemia directly inhibits the proliferation of T lymphocytes in Gut-associated lymphoid tissue (GALT), leading to a decrease in the synthesis and secretion of secretory immunoglobulin A (SIgA), making it difficult to effectively clear opportunistic pathogens from the mucosal surface (Jia et al., 2020; Cui H. et al., 2023). Shen et al., in an animal experiment using HF rats, further investigation in HF rats revealed downregulation of the anti-inflammatory factor interleukin-10 (IL-10) and elevated levels of the pro-inflammatory factor tumor necrosis factor-*α* (TNF-α) in lamina propria lymphocytes of the intestinal mucosa. This immune imbalance further exacerbated the dysbiosis (Shen et al., 2025). Perticone M et al., in a clinical observational study involving multidimensional sample analysis of 120 HF patients and 40 healthy controls further confirmed that the inhibition of the intestinal immune barrier by HF is a multi-level pathological process resulting from the imbalance of immune cell subset polarization, leading to dual impairment of SIgA synthesis and transport, and consequently, immune factor dysbiosis (Perticone et al., 2024).

#### 2.1.3 Indirect effects

In the pathological process of HF, excessive activation of the sympathetic nervous system is a key indirect factor driving the imbalance of gut microbiota homeostasis (Figure 1C). Fountoulakis et al. (2025) in a cohort study of HF patients, they found that systemic chronic inflammation induced by sympathetic nerve activation significantly inhibited the proliferation of beneficial bacteria producing SCFAs in the gut, reducing their abundance by 30–40% compared to healthy individuals. This reduction in these bacteria directly weakens the nutritional support and anti-inflammatory effects of SCFAs on the intestinal epithelium, further disrupting the microbial imbalance. Liu et al. in a chronic heart failure (CHF) rat model, reverse validation showed that butyrate, a metabolite of intestinal microbiota, can reduce sympathetic nerve activation by inhibiting inflammation of paraventricular microglia. Conversely, this suggests that sympathetic activation in HF can inhibit butyrate-producing bacteria abundance through central inflammatory feedback, forming a harmful cycle and leading to a significant decline in microbial metabolic function (Liu et al., 2024). In addition, Shu et al. proposed a mechanism of sympathetic nerve-driven changes in intestinal immune factors can be compared to HF. Although this study was not conducted in a HF model, the proposed mechanism may be extrapolated to HF. Sympathetic activation in HF inhibits the production and release of IL-22 in the intestinal mucosa, and the reduction of IL-22 disrupts the integrity of the intestinal barrier, providing enrichment conditions for hypoxia-resistant opportunistic pathogens, further exacerbating the dysbiosis of the gut microbiota (Shu et al., 2025). As reviewed by Wang et al. in their study of ejection fraction-preserving HF, they further pointed out that sympathetic nerve-mediated intestinal microarterial contraction further reduces intestinal blood flow, which, combined with the intestinal ischemia state of HF itself, leads to a deterioration of the hypoxic microenvironment in the intestine, thereby inhibiting the colonization of aerobic-sensitive beneficial bacteria (Wang and Xiao, 2022).

HF patients need to use multiple cardiovascular drugs in combination for a long time to control their condition, and these drugs may further aggravate the dysbiosis on the basis of intestinal ischemia and barrier damage, becoming an important secondary inducing factor (Figure 1C). In a hypertensive rat model, diuretic-induced hypokalemia was shown to lead to intestinal pH acidification, while fluid loss reduced the substrates for gut microbiota fermentation, thus doubly inhibiting the abundance of SCFA-producing beneficial bacteria and promoting the proliferation of acid-resistant pathogens (Jaworska et al., 2017). Although this study was not conducted in a HF-specific model, the observed mechanisms are considered relevant to HF patients who commonly receive long-term diuretic therapy. Clinical observational studies in hypertensive cohorts suggest that angiotensin-converting enzyme inhibitors (ACEIs) may slightly increase the abundance of *Enterococcus* through non-specific inhibition of the intestinal ACE2 parapathway (Li J. et al., 2024; Penninger et al., 2021). While direct evidence in HF populations is limited, the effects of ACE2 modulation on intestinal microbiota are considered relevant given the widespread use of ACEIs in HF management. Evidence from hypertensive models and cohorts suggests that angiotensin receptor blockers (ARBs) may occasionally lead to a relative decrease in Bacteroidetes, although the individual effect is relatively mild, it can be superimposed on the already reduced gut microbiota diversity in HF, exacerbating the microbiota imbalance. These findings require validation in dedicated HF populations. Preclinical studies suggest that beta-blockers may slow intestinal peristalsis by inhibiting sympathetic nerve activity, leading to food residue retention and exacerbating the excessive proliferation of gas-producing fermenting bacteria such as *Enterococcus* (Muller et al., 2020). However, the direct impact of beta-blockers on gut microbiota composition in HF patients has not been comprehensively investigated. In a non-HF metabolic disorder context, statins have been shown to inhibit the abundance of Glucagon-like peptide-1 (GLP-1)-producing bacteria in the gut and reduce intestinal GLP-1 secretion (She et al., 2024). Whether this effect is reproduced in HF populations remains to be confirmed, although it is plausible given the frequent comorbidity of metabolic disorders in HF. Limited preclinical evidence suggests that long-term aspirin use may mildly irritate the damaged intestinal mucosa in HF patients. Combined with intestinal barrier dysfunction, this could lead to a slight increase in the abundance of Proteobacteria, increasing the risk of microbiota translocation (Roberts et al., 2018). Observational data from critically ill patients suggest that warfarin may inhibit the proliferation of vitamin K2-producing bacteria in the gut, such as *Bacteroides fragilis* and *Enterococcus faecalis* (Schött et al., 2021). In HF patients, who often have inadequate vitamin K intake and absorption, this effect could further weaken the gut microbiota’s ability to synthesize vitamin K, potentially exacerbating related gut microbiota dysfunction. However, direct evidence from HF-specific studies is currently lacking.

### 2.2 The pathological mechanism of gut microbiota exacerbating HF

#### 2.2.1 Imbalance of metabolites

Gut microbiota regulate host physiological functions by producing a variety of bioactive metabolites. In HF, this regulatory balance is disrupted, manifested as the excessive production of propathogenic metabolites and the significant loss of protective metabolites, jointly driving the progression of HF (Figure 1D).

##### 2.2.1.1 Toxic effects of HF-promoting metabolites

TMAO is a key propathogenic metabolite in the reverse regulation of HF by gut microbiota. Its production depends on the conversion of precursors such as choline and L-carnitine into trimethylamine (TMA) by gut microbiota, which is then catalyzed by flavin monooxygenase 3 (FMO3) in the liver. A clinical cohort study (*n* = 253) reported that FMO3-mediated TMAO production pathway is significantly associated with poor clinical outcomes in HF, especially heart failure with reduced ejection fraction (HFrEF) (Zhang Y. et al., 2021; Wei et al., 2022a). TMAO exacerbates HF progression through multiple pathways, primarily involving inflammatory activation, oxidative stress, and myocardial remodeling. In preclinical studies, TMAO has been shown to activate the protein kinase C (PKC)/NF-κB pathway, promoting the release of TNF-*α* and IL-6, and amplifies systemic inflammation by disrupting the intestinal barrier and increasing endotoxin entry into the bloodstream (Wang Z. et al., 2024; Hemmati et al., 2023; Cienkowski et al., 2024). At the same time, it induces excessive reactive oxygen species (ROS) production in cardiomyocytes and inhibits antioxidant enzyme activity, triggering caspase-mediated apoptosis (Yafarova et al., 2024; Li et al., 2019a). In terms of myocardial remodeling and cardiorenal interaction, animal studies suggest that TMAO promotes excessive myocardial collagen deposition and increases myocardial stiffness by upregulating transforming growth factor-*β* (TGF-β1) and matrix metalloproteinases/Tissue inhibitors of metalloproteinases (MMPs/TIMP) expression imbalance (Florek et al., 2025; Salzano et al., 2022). Furthermore, as proposed in a recent review, TMAO, as a bridging molecule in cardiorenal syndrome, reduces water and sodium excretion by impairing renal osmolarity regulation, thus increasing cardiac volume load (Zhang J. et al., 2023).

Imidazole propionate (ImP) is a pro-HF metabolite generated by the metabolism of dietary L-histidine by gut microbiota harboring the enzyme urocanate reductase. In two large, independent clinical cohorts (a European population-based cohort, *n* = 1,985; and a North American hospital-based cohort, *n* = 2,155), Molinaro et al. demonstrated that circulating ImP levels were independently associated with reduced ejection fraction and HF, even after adjusting for traditional cardiovascular risk factors. Moreover, elevated ImP (highest quartile) was an independent predictor of 5-year all-cause mortality (Molinaro et al., 2023). This clinical association was further corroborated by Raju et al. (2024), who showed in an independent HF cohort that ImP levels correlated with markers of intestinal barrier dysfunction and disease severity. Regarding the underlying pathogenic mechanisms, preclinical and *in vitro* studies suggest that ImP activates the mammalian target of rapamycin complex 1 (mTORC1) while suppressing Adenosine Monophosphate-Activated Protein Kinase (AMPK), leading to impaired myocardial energy metabolism and inhibition of autophagy. Additionally, ImP has been reported to activate the NF-κB/NLRP3 inflammasome axis and induce ROS accumulation, and to promote myocardial fibroblast proliferation through the TGF-β1/Smad pathway, thereby contributing to myocardial remodeling (Zeng et al., 2025).

Beyond TMAO and ImP, recent studies have identified several additional gut microbiota-derived metabolites that contribute to HF pathogenesis. Phenylacetylglutamine (PAGln) is produced when gut bacteria metabolize dietary phenylalanine to phenylacetic acid (PAA), which is subsequently conjugated with glutamine in the liver. Plasma PAGln levels are markedly elevated in HF patients and independently associated with all-cause mortality. Clinical cohort studies have shown that plasma PAGln levels are markedly elevated in HF patients and independently associated with all-cause mortality (Tang et al., 2024; Romano et al., 2023). Mechanistically, animal experiments suggest that PAGln exerts negative inotropic effects primarily through β2-adrenergic receptor (β2AR) signaling, attenuating sympathetically driven enhancement of myocardial contractility, while directly upregulating brain natriuretic peptide (BNP) gene expression and increasing cardiac load. Notably, the precursor PAA itself possesses independent pathogenic properties. In animal and in vitro studies, PAA drives excessive mitochondrial H₂O₂ production through NADPH oxidase 4 (NOX4) activation, triggering endothelial cell DNA damage and a senescence phenotype. The ensuing senescence-associated secretory phenotype (SASP) releases pro-inflammatory factors, establishing a persistent vascular inflammatory microenvironment (Saeedi Saravi et al., 2025; Chen et al., 2024). Clinical studies indicate that Indoxyl sulfate (IS) and p-cresyl sulfate (PCS) are uremic toxin-class metabolites that directly damage cardiomyocytes through oxidative stress and pro-inflammatory responses. Their pathogenic significance is particularly pronounced in the context of cardiorenal syndrome (CRS), where impaired renal clearance leads to their accumulation (Imazu et al., 2017; Vanholder et al., 2014). In addition, N,N,N-trimethyl-5-aminovaleric acid (TMAVA) inhibits carnitine synthesis and uptake by blocking *γ*-butyrobetaine hydroxylase and organic cation/carnitine transporter 2 (OCTN2), thereby suppressing myocardial fatty acid oxidation and resulting in lipid accumulation and mitochondrial dysfunction. TMAVA has been identified as an independent cardiovascular toxic molecule in preclinical studies (Zhao M. et al., 2022).

##### 2.2.1.2 Protective loss of anti-inflammatory metabolites

SCFAs (mainly acetic acid, propionic acid, and butyric acid) are the key protective molecules in the gut microbiota’s metabolism of dietary fiber. The abundance of SCFA-producing bacteria is reduced in HF patients, leading to a decrease in SCFA levels with increasing NYHA grade, thus weakening their protective effect on the myocardium (Zhao P. et al., 2022; Modrego et al., 2023; Chulenbayeva et al., 2025). From an anti-inflammatory perspective, preclinical studies indicate that SCFAs may inhibit the NF-κB pathway by activating GPR41/GPR43 receptors, thereby reducing the release of pro-inflammatory factors (Kondapalli et al., 2025; Wang A. et al., 2023; Du et al., 2025). Butyrate can inhibit NLRP3 inflammasome assembly (Xu et al., 2025). In animal experiments, propionic acid promotes regulatory T cell differentiation, inhibits macrophage M1 polarization, and reduces myocardial inflammatory infiltration (Hemmati et al., 2023; Bartolomaeus et al., 2019). In terms of improving myocardial energy metabolism, butyrate promotes mitochondrial biosynthesis and inhibits its fission through the Aryl hydrocarbon receptor pathway, maintaining mitochondrial function (Wang A. et al., 2023; Du et al., 2025; Xu et al., 2025). Acetate and propionate maintain myocardial energy homeostasis by regulating myocardial substrate preference, optimizing glycolipid metabolic balance and improving insulin sensitivity (Zhao P. et al., 2022; Yu et al., 2023; Pakhomov and Baugh, 2021).

Secondary bile acids are generated by the conversion of primary bile acids by gut microbiota, mainly through the farnesoid X receptor (FXR) and takeda g protein-coupled receptor 5 (TGR5). In a clinical cohort study of 142 patients with chronic HF, Mayerhofer et al. reported that bile acid metabolism was significantly altered, with an increased secondary-to-primary bile acid ratio, and that this alteration was negatively correlated with left ventricular ejection fraction (LVEF), suggesting that bile acid dysmetabolism is a clinically relevant feature of HF progression (Mayerhofer et al., 2017). Mechanistically, animal experiments and *in vitro* studies have demonstrated that secondary bile acid binding to FXR inhibits NF-κB pathway transcriptional activity (Tang et al., 2017; Zhou et al., 2020; Du J. et al., 2020). TGR5 induced M2 polarization and inhibited NLRP3 inflammatory bodies in macrophages (Escobar et al., 2025b; Williams et al., 2025). In addition, secondary bile acids can enhance the expression of tight junction proteins in the intestinal barrier and reduce LPS entry into the bloodstream (Branchereau et al., 2019; Jin et al., 2019; Song et al., 2022). In a randomized, double-blind, placebo-controlled crossover trial (*n* = 17), von Haehling et al. demonstrated that ursodeoxycholic acid (UDCA) supplementation improved peripheral blood flow in patients with chronic HF, providing direct interventional evidence supporting the therapeutic potential of bile acid modulation in this population (von Haehling et al., 2012). At the level of regulating energy metabolism, FXR improves myocardial lipid metabolism by inhibiting SREBP-1c and promoting peroxisome proliferator-activated receptor *α*. TGR5 promotes mitochondrial biosynthesis by upregulating peroxisome proliferator-activated receptor gamma coactivator 1α (PGC-1α), and the two work together to maintain myocardial energy homeostasis (Escobar et al., 2025a; Williams et al., 2025).

Indole and its derivatives represent another class of protective products generated by gut microbial metabolism of dietary tryptophan. Among these, indole-3-propionic acid (IPA) has attracted considerable attention for its cardiovascular protective properties. IPA binds the aryl hydrocarbon receptor (AhR), inducing nuclear translocation and upregulating SIRT3 expression, which enhances mitochondrial antioxidant capacity and improves diastolic function (Wang Y. C. et al., 2024). Indole-3-aldehyde (IAld) primarily reinforces intestinal epithelial barrier integrity and tight junction protein expression through the AhR–IL-22 pathway, thereby reducing the translocation of harmful molecules such as LPS at the source and indirectly protecting the myocardium from inflammatory damage (Zelante et al., 2013). As a gaseous signaling molecule, H₂S is partly regulated by sulfate-reducing bacteria (e.g., *Desulfovibrio*) in the gut microbiota. In HF, the cystathionine-*γ*-lyase (CSE)/H₂S signaling axis is impaired, leading to insufficient H₂S production. H₂S exerts dual antioxidant effects through direct ROS scavenging and upregulation of MnSOD/Cu/Zn-SOD, and promotes nuclear factor erythroid 2-related factor 2 (NRF2) nuclear translocation via S-sulfhydration of KEAP1, thereby activating the SLC7A11/GSH/GPx4 axis to inhibit ferroptosis (Zhang H. et al., 2024). Polyphenol metabolites and vitamins (particularly vitamin D) can also exert protective effects via the gut-heart axis; however, their bioavailability is often substantially limited by HF-associated intestinal dysfunction, including reduced absorption and decreased microbial transformation efficiency (Guo S. et al., 2025).

In addition to the metabolites discussed in detail above, the gut microbiota produces a range of other bioactive molecules involved in the positive or negative regulation of HF, spanning pathological processes including inflammation, oxidative stress, energy metabolism, and fibrosis. To provide a systematic overview of the associated bacterial genera, target pathways, mechanisms of action, and evidence in HF for these metabolites, we have summarized the relevant information in Table 1.

**Table 1:** Gut microbiota-derived metabolites implicated in HF pathogenesis.

Metabolite	Key producing bacteria	Primary receptors/targets	Mechanisms in HF	Evidence in HF	References
Deleterious metabolites					
TMAO	Firmicutes (clostridia), Proteobacteria (*Proteus*, *Escherichia*), Actinobacteria	PKC/NF-κB, TGF-β/Smad3, NLRP3, Nrf2, SIRT1, Piezo1	Activates PKC/NF-κB promoting TNF-α/IL-6 release; induces ROS via SIRT1 inhibition and mitochondrial dysfunction; promotes cardiac fibrosis through TGF-β1/Smad3; disrupts calcium homeostasis and electrophysiological stability; impairs renal water-sodium handling	Independently predicts all-cause mortality/hospitalization in HF; FMO3-TMAO axis correlates with poor outcomes in HFrEF (Asian cohort, *n* = 253)	Wei et al. (2022b), Wang J. et al. (2024), and Yafarova et al. (2024)
ImP	*Streptococcus mutans*, *Aerococcus urinae*, and other UrdA-harboring species	mTORC1, AMPK, NF-κB/NLRP3, TGF-β1/Smad	Activates mTORC1 while inhibiting AMPK, causing energy metabolism reprogramming and autophagy suppression; triggers NF-κB/NLRP3-mediated inflammation; promotes cardiac fibroblast proliferation via TGF-β1/Smad; inhibits angiogenesis	Serum ImP elevated in HF (*n* = 1,606), correlating with LV dysfunction, NT-proBNP, and 5-year mortality; linked to intestinal barrier dysfunction markers	Molinaro et al. (2023) and Raju et al. (2024)
PAGln	*Clostridium*, *Bacteroides*, *Enterococcus*	*β*₂-AR, CaMKII, NOX2, TLR4/AKT/mTOR	β₂-AR-mediated negative inotropy inhibiting sympathetic contractility enhancement; upregulates BNP gene expression; induces oxidative stress and mitochondrial fragmentation; promotes atrial fibrosis via ferroptosis and NLRP3 activation	Independently correlates with 5-year all-cause mortality in stable HF (Cleveland cohort *n* = 1,043, Berlin cohort *n* = 833); BNP r = 0.924 (*p* < 0.001), LVEF r = −0.587 (*p* < 0.001)	Tang et al. (2024), Romano et al. (2023), Zhang Z. et al. (2023), and Fu et al. (2023)
PAA	*Bacteroides fragilis*, *Clostridium* spp.	NOX4, CaMKII/HDAC4, SASP	Activates NOX4 to enhance mitochondrial H₂O₂, triggering DNA damage and endothelial cell senescence; induces SASP-mediated persistent vascular inflammation; epigenetically dysregulates pro-inflammatory genes via CaMKII-HDAC4; inhibits mitochondrial respiration and glycolysis	Elevated PAA independently associated with increased 30-day mortality in acute decompensated HF (*n* = 167)	Saeedi Saravi et al. (2025) and Chen et al. (2024)
IS	*Escherichia coli*, *Clostridium* spp.	ERK/p38 MAPK, NF-κB, AhR	Activates ERK/p38 MAPK and NF-κB inducing oxidative stress and inflammation; inhibits NOS activity; downregulates erythropoietin and Klotho; promotes cardiomyocyte hypertrophy and fibrosis; impairs contractile function	Plasma IS elevated in CHF (1.38 ± 0.84 vs. 0.12 ± 0.07 μg/mL, *p* < 0.001); IS levels correlate with cardiac function (FS) and predict CV events in mild CHF (5-year follow-up, *n* = 165)	Imazu et al. (2017), Imazu et al. (2020), and Lin et al. (2015)
PCS	*Clostridioides difficile*, *Blautia hydrogenotrophica*, *Olsenella uli*, *Romboutsia lituseburensis*	NADPH oxidase, MAPK	Induces NADPH oxidase activation and ROS generation; promotes cardiomyocyte apoptosis (Bax↑, Bcl-2↓); causes diastolic dysfunction (E/A ratio alteration); accumulates in CRS due to reduced renal clearance	PCS promotes cardiac apoptosis and diastolic dysfunction in 5/6 nephrectomy mice; PCS is an independent predictor of CV mortality in CKD patients on hemodialysis	Han et al. (2015)
TMAVA	Not fully characterized at genus level	γ-butyrobetaine hydroxylase (BBH), OCTN2	Inhibits BBH disrupting carnitine synthesis; blocks OCTN2-mediated myocardial carnitine uptake; inhibits fatty acid oxidation causing lipid accumulation, mitochondrial dysfunction, and ROS overproduction; induces cardiac hypertrophy	Plasma TMAVA elevated in HF patients (metabolomics, *n* = 1,647); TMAVA-treated mice develop cardiac hypertrophy with reduced contractility and exercise intolerance	Zhao M. et al. (2022)
LPS (microbial structural component)	*Escherichia coli*, *Klebsiella*, *Enterobacter*	TLR4/MyD88/NF-κB, NLRP3, STING-IRF3, CaSR	Binds TLR4 activating MyD88/NF-κB cascade; triggers NLRP3 inflammasome-mediated pyroptosis via caspase-1/GSDMD; disrupts calcium cycling via CaSR-PLCβ-IP₃; activates ferroptosis via NCOA4-dependent ferritin degradation	Serum LPS inversely correlated with cardiac function in CHF patients (*n* = 20); elevated in HF patients with intestinal congestion	Sandek et al. (2012)
Protective metabolites					
Acetate (SCFA)	*Bifidobacterium*, *Lactobacillus*, *Bacteroides*, *Prevotella*, *Blautia*	GPR41, GPR43, TGF-β1/Smad2	Activates GPR41/GPR43 inhibiting TGF-β1/Smad2 phosphorylation, blocking fibroblast-to-myofibroblast transformation; reduces sympathetic tone; remodels gut microbiota; modulates RAAS activity	High-fiber diet + acetate supplementation prevents hypertension and HF development in DOCA-salt hypertensive mice; acetate reduces heart rate and cardiac contractility via GPR41/43	Marques et al. (2017), Poll et al. (2021), and Lin et al. (2022)
Propionate (SCFA)	*Bacteroides*, *Phascolarctobacterium*, *Dialister*, *Veillonella*	GPR41; caveolin-1/ACE2 axis	Activates GPR41 inhibiting Ang II-caveolin-1 interaction, upregulating ACE2 to mitigate I/R injury; reduces myocardial oxidative stress and inflammation	Propionate alleviates myocardial I/R injury in Ang II-treated mice via GPR41-ACE2 axis	Deng et al. (2022)
Butyrate (SCFA)	*Faecalibacterium prausnitzii*, *Roseburia*, *Eubacterium rectale*, *Coprococcus*, *Bacteroides vulgatus*	GPR41/43/109A, HDAC, NF-κB/STAT, PGC-1α/TFAM, TGF-β/Smad	Inhibits NLRP3 inflammasome; suppresses M1 and promotes M2 macrophage polarization via NF-κB/STAT; enhances mitochondrial biogenesis through PGC-1α/TFAM via AMPK; inhibits fibroblast activation via TGF-β/Smad; acts as HDAC inhibitor upregulating anti-fibrotic genes	*Bacteroides vulgatus* alleviates HF via butyric acid in TAC mice; butyrate exerts inotropic effects with vasorelaxant and cardioprotective properties in isolated rat hearts	Du et al. (2025), Seefeldt et al. (2024), and Li X. et al. (2022)
Secondary bile acids (HDCA, UDCA, LCA, DCA)	*Clostridium scindens*, *Clostridium hylemonae*, *Lactobacillus*, *Bifidobacterium*	FXR, TGR5 (GPBAR1)	FXR activation represses NF-κB and endothelin-1 transcription; TGR5 induces M2 macrophage polarization and suppresses NLRP3; enhances intestinal barrier tight junctions reducing LPS translocation; FXR/TGR5 regulate lipid metabolism and mitochondrial biogenesis; UDCA inhibits fibroblast-to-myofibroblast transition	Altered secondary/primary BA ratio in chronic HF (*n* = 142); UDCA improves peripheral hemodynamics in CHF (randomized crossover trial, *n* = 17); CDCA/DCA induce positive inotropic effects in isolated rat hearts	Mayerhofer et al. (2017), von Haehling et al. (2012), Gao et al. (2021), and Reilly-O'Donnell et al. (2024)
IPA (indole derivative)	*Clostridium sporogenes*, *Peptostreptococcus anaerobius*	AhR, SIRT3, NAD^+^ salvage pathway, HDAC6/NOX2, PI3K/AKT/GSK3β	AhR binding upregulates SIRT3 enhancing mitochondrial antioxidant capacity; activates NAD^+^ salvage pathway optimizing energy metabolism; suppresses HDAC6/NOX2 reducing inflammation; activates PI3K/AKT/GSK3β reducing apoptosis; inhibits NF-κB/NLRP3 inflammasome; promotes macrophage cholesterol efflux via ABCA1	IPA protects against HFpEF in mice (improved diastolic function, reduced fibrosis); IPA deficiency causally related to atherosclerotic CVD (Mendelian randomization)	Wang Y. C. et al. (2024), Xue et al. (2022), Li C. et al. (2024), and Zhang et al. (2024b)
IAld, IAA (indole derivatives)	*Lactobacillus reuteri*, *Bacteroides*	AhR, IL-22 signaling	AhR activation induces IL-22 production reinforcing intestinal epithelial barrier and tight junction expression; inhibits lipid/protein oxidation preserving myocardial membrane integrity; reduces I/R-induced oxidative damage	Indole derivatives reduce I/R-induced myocardial infarct size in rabbit models; AhR-IL-22 pathway maintains intestinal barrier (preclinical evidence)	Li Q. et al. (2022) and Zelante et al. (2013)
H₂S	*Desulfovibrio*, *Bilophila wadsworthia*, host CSE also contributes	KEAP1/NRF2, SLC7A11/GSH/GPx4, AMPK, PI3K/AKT/eNOS, SIRT3/TGF-β1, SERCA2a/MuRF1	Direct ROS scavenging + MnSOD/Cu/Zn-SOD upregulation; KEAP1 S-sulfhydration promotes NRF2-SLC7A11/GSH/GPx4 axis inhibiting ferroptosis; SIRT3 S-sulfhydration suppresses TGF-β1/Smad2/3 reducing fibrosis; MuRF1 S-sulfhydration restores SERCA2a function improving calcium handling; inhibits sympathetic-RAAS overactivation; suppresses TLR4/NLRP3	H₂S donors improve cardiac function in pressure-overload HF mice; NaHS reverses HFpEF phenotype in HFD + L-NAME mice; CSE/H₂S axis impaired in HF	Polhemus et al. (2013), Huang et al. (2023), Zhang H. et al. (2024), and Peng et al. (2022)
Polyphenol metabolites	*Lactobacillus*, *Bifidobacterium* (hydrolysis); *Eggerthella lenta* (dehydroxylation)	Keap1/Nrf2/ARE, NF-κB, MAPK/JNK, PPARγ, AMPK, SERCA2a	Direct ROS/RNS scavenging; Keap1/Nrf2/ARE activation upregulating antioxidant enzymes; NF-κB/MAPK/JNK inhibition suppressing inflammation; mitochondrial membrane stabilization and mPTP inhibition; AMPK activation promoting fatty acid oxidation; SERCA2a activation maintaining calcium homeostasis	Urolithin B-glucuronide protects cardiomyocytes from TMAO-induced impairment (*in vitro*); >90% of polyphenols require microbial conversion for bioavailability, reduced in HF dysbiosis	Savi et al. (2018) and Marques et al. (2017)
Vitamin D	N/A (host-derived; Absorption impaired by HF-associated intestinal edema)	VDR, SOD/catalase, SERCA2a/PLB, TGF-β, NF-κB, Drp1/PINK1	Enhances SOD/catalase activity reducing ROS/MDA; upregulates mitochondrial biogenesis regulators; optimizes calcium channel kinetics and SERCA2a/PLB ratio; inhibits TGF-β/NF-κB pathways reducing fibrosis and inflammation; reverses Drp1/PINK1 downregulation restoring mitophagy	Low 25(OH)D increases all-cause mortality and HF readmission; high-dose VD₃ (4,000 IU/d, 12 months) enhances LVEF and reduces ventricular volume in CHF (RCT, VINDICATE, *n* = 229)	Witte et al. (2016), Shahidi et al. (2024), and Zhang et al. (2018)
Vitamin C	N/A (host-derived; Absorption impaired by HF-associated intestinal edema)	ROS scavenging, eNOS/NO pathway	Directly scavenges free radicals and lipid peroxidation products; improves endothelium-dependent vasodilation partly via NO; suppresses neutrophil superoxide anion production reducing inflammatory oxidative damage	Plasma VC inversely associated with HF risk (British Regional Heart Study, *n* = 3,919); acute IV VC (2 g) enhances platelet NO responsiveness and lowers oxidative stress in CHF patients	Wannamethee et al. (2013) and Morelli et al. (2020)

ACE2, Angiotensin-converting enzyme 2; AhR, Aryl hydrocarbon receptor; Ang II, Angiotensin II; CaSR, Calcium-sensing receptor; eNOS, Endothelial Nitric Oxide Synthase; ERK, Extracellular signal-regulated kinase; GPR41/43, G-protein coupled receptor 41/43; GSDMD, Gasdermin D; HDAC, Histone deacetylase; IAA, indole-3-acetic acid; MAPK, Mitogen-activated protein kinase; MyD88, Myeloid differentiation primary response 88; RAAS: Renin-angiotensin-aldosterone system; SERCA2a, Sarco/endoplasmic reticulum Ca2 + -ATPase; PI3K, Phosphatidylinositol 3-Kinase. (1) Some metabolites show dose-dependent dual effects; classification is based on their overall effect in HF. (2) All references listed are original research articles (clinical cohort studies, RCTs, or experimental studies); review articles have been excluded to ensure evidence quality. (3) Bacterial associations are based on current evidence from metagenomic and culture-based studies.

#### 2.2.2 Activation of inflammatory pathways

##### 2.2.2.1 LPS activates the toll-like receptor 4 (TLR4) pathway

LPS released by intestinal Gram-negative bacteria is a key molecule in gut microbiota-mediated HF inflammation. In HF, impaired intestinal barrier function leads to LPS entry into the bloodstream. LPS forms a complex with serum lipopolysaccharide-binding protein (LBP), which efficiently binds to TLR4 receptors on the surface of cardiomyocytes and immune cells (Figure 1E). Upon activation of the TLR4 receptor, downstream signaling molecules are rapidly recruited via the MyD88 pathway, ultimately activating the lkB Kinase complex, leading to the degradation of IκB*α* and the release of NF-κB. Activated NF-κB then enters the nucleus and initiates the transcription of pro-inflammatory factors such as TNF-α, IL-6, and IL-1β (Zhang X. et al., 2025; Cui Y. et al., 2023). *In vitro* and *in vivo* experiments demonstrated that serum LPS levels and expression of key proteins in the TLR4/NF-κB pathway in myocardial tissue were significantly upregulated in HF model mice (Zhang X. et al., 2025). The stepwise release of these pro-inflammatory factors contributes to myocardial cell apoptosis, induces oxidative stress, and activates myocardial fibroblasts to promote collagen deposition. At the same time, inflammation-mediated vascular endothelial damage exacerbates myocardial ischemia, jointly accelerating ventricular remodeling (Zhang C. et al., 2025).

##### 2.2.2.2 NLRP3 Inflammatory amplifies inflammation

The NLRP3 inflammasome is a key mechanism for amplifying microbiome-mediated inflammation (Figure 1E). Dysbiosis-induced release of LPS and TMAO provides the “initiation signal”, promoting the transcription of NLRP3 and cytokine precursors via the TLR4/NF-κB pathway. Simultaneously, dysbiosis-induced ROS accumulation and potassium efflux in cardiomyocytes constitute the “activation signal”, triggering the assembly of NLRP3 with Apoptosis-associated speck-like protein containing a CARD (ASC) and caspase-1 to form a functional inflammasome complex (Lu et al., 2022; Fusco et al., 2020). Activated caspase-1 cleaves precursors to generate active IL-1β/IL-18, recruiting inflammatory cells to infiltrate the myocardium; it also cleaves GSDMD to induce pyroptosis in cardiomyocytes. The cellular contents (Damage-associated molecular patterns, DAMPs) released during pyroptosis further activate NLRP3 in surrounding cells, forming a continuously amplified inflammatory cycle (Wu C. et al., 2025; Skalny et al., 2025). NLRP3 pathway activation is significant in the myocardial tissue of HF model mice. In animal models, knockout of NLRP3 can significantly improve cardiac function (Lu et al., 2022). This over-activated inflammatory response can also damage mitochondrial function, interfere with energy metabolism, and accelerate the progression of HF (Fountoulakis et al., 2025; Yin et al., 2025).

#### 2.2.3 Synergistic damage of the gut-immune-cardiac axis

Gut microbiota dysbiosis and intestinal barrier damage constitute a key pathway for the synergistic damage of the “gut-immune-heart” axis by activating local immune cells in the gut and promoting their migration and infiltration into the myocardium (Figure 1F) (Yu et al., 2025; Carrillo-Salinas et al., 2020). Local intestinal immunity is preferentially activated. LPS, metabolic intermediates, and leaked nutrients released by the gut microbiota bind to pattern recognition receptors on the surface of immune cells, initiating activation: CD4^+^ T cells differentiate into Th1/Th17 pro-inflammatory phenotypes, and Treg function is suppressed; intestinal macrophages polarize into the M1 type and release IL-1β and TNF-α; mucosal-associated invariant T cells exhibit abnormal activation signals due to gut microbiota dysbiosis (Carrillo-Salinas et al., 2020; Wang J. et al., 2025). Subsequently, immune signals spread throughout the body and infiltrate the myocardium, releasing pro-inflammatory factors that diffuse through the bloodstream and activate local myocardial inflammatory pathways (Stanciu, 2019; Madhur et al., 2021). On the other hand, they directly migrate to the myocardium to participate in immune damage. LPS entry into the bloodstream activates the differentiation of monocytes throughout the body into M1 macrophages, and under the action of chemokines released from the myocardium, they migrate and infiltrate the myocardium in a directed manner (Gu et al., 2023; Deng et al., 2024). Gut-derived antigen stimulation and myocardial chemotactic signals jointly promote T cell infiltration. In addition, gut microbiota metabolites can also enhance the migration ability of immune cells by regulating the expression of immune cell adhesion molecules (Madhur et al., 2021; Farini et al., 2023). This ultimately leads to persistent myocardial damage and remodeling. The infiltrated M1 macrophages and Th1/Th17 cells release a large number of pro-inflammatory factors locally in the myocardium, inducing myocardial cell apoptosis/pyroptosis, activating fibroblasts and promoting collagen deposition. Meanwhile, immune damage can induce myocardial microcirculatory disorders, impair mitochondrial function, exacerbate energy metabolism disorders, and promote the progression of HF (Yu et al., 2025; Quagliariello et al., 2025; Liu R. et al., 2025; Zhuang L. et al., 2023).

## 3 Modern medicine’s treatment of HF based on gut microbiota

**Figure 2:**
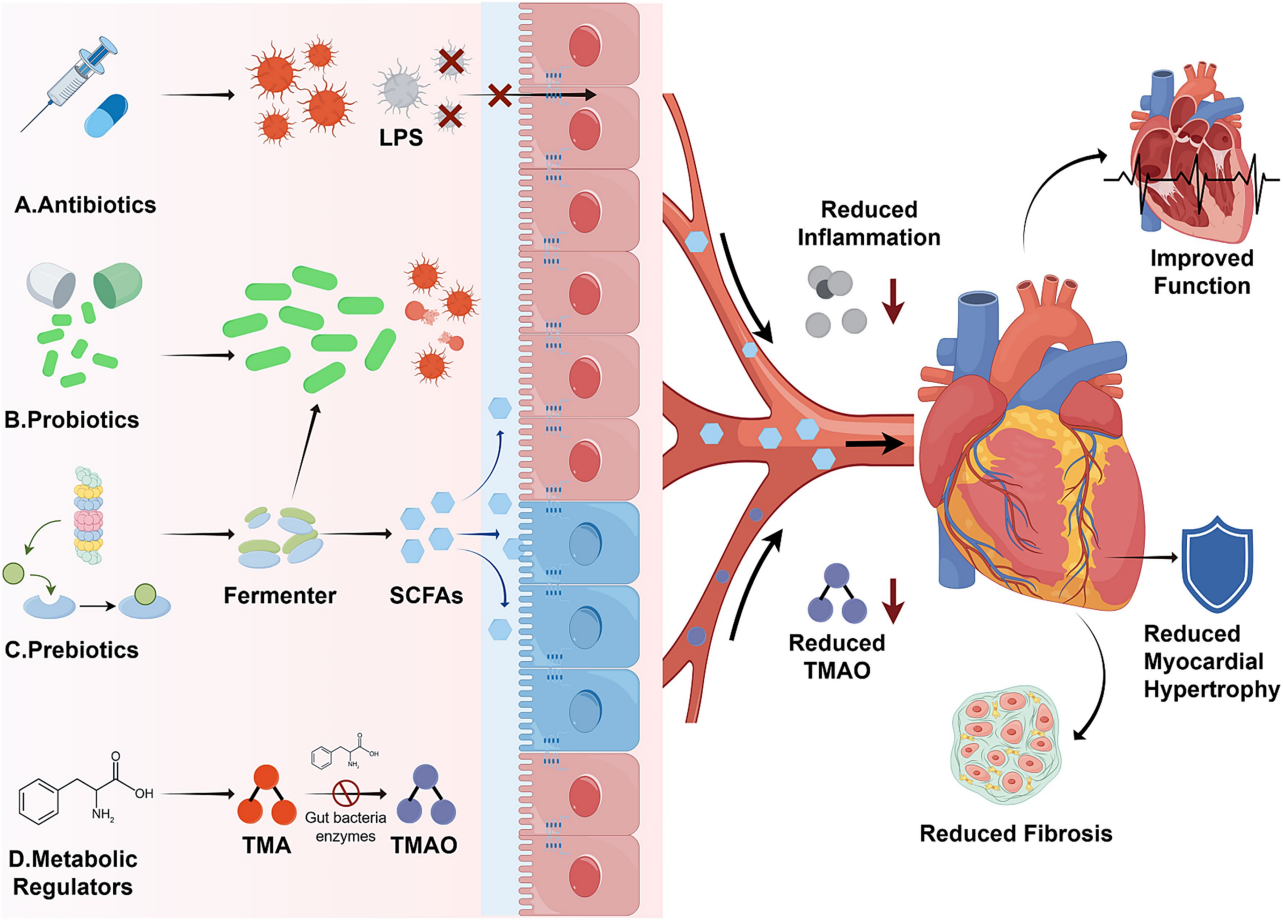
Mechanism of western medicine regulating gut microbiota in treating HF (gut-heart axis) (by Figdraw).

Traditional treatments for HF revolve around the neurohormonal axis as the core of intervention, and have achieved evidence-based results in reducing hospitalization and mortality rates. However, the direct impact of these drugs on the gut microbiota is extremely limited, and even produces negative effects. In recent years, with the increasing attention paid to the role of gut microbiota in HF, research on Western medicines to improve HF by directly or indirectly regulating gut microbiota is gradually being carried out. Currently, Western medicines used to regulate gut microbiota are mainly divided into four categories, including antibiotics, probiotics, prebiotics, and microbiota metabolism regulators. Some drugs have shown therapeutic potential for HF in clinical or animal experiments (Figure 2).

### 3.1 Antibiotics

Antibiotics alleviate enterogenic inflammation associated with HF by selectively inhibiting the proliferation of harmful intestinal bacteria, reducing endotoxin accumulation, and decreasing the production of pro-inflammatory metabolites. Among these drugs, selective intestinal decontamination (SID) is a hot research topic, with typical drugs including polymyxin B and tobramycin. A pilot trial conducted by Conraads et al. in patients with advanced CHF showed that oral polymyxin B and tobramycin significantly reduced intestinal toxin levels, monocyte CD14 expression, and pro-inflammatory cytokine concentrations (Nagatomo and Tang, 2015). Rifaximin, a non-absorbable antibiotic, is used to regulate the gut microbiota of HF patients. Although a pilot RCT (*n* = 151) did not show significant improvement in LVEF or TMAO levels (Awoyemi et al., 2021), preclinical studies suggest that rifaximin may promote the proliferation of beneficial bacteria such as *Bifidobacteria* and *Lactobacillus*, improving gut microbiota composition and barrier function (Ponziani et al., 2017). In addition, rifaximin can directly reduce local intestinal inflammation and decrease the risk of endotoxemia caused by LPS entering the bloodstream by inhibiting NF-κB pathway activation. However, the efficacy of antibiotics in regulating the gut microbiota varies significantly among individuals, and the course of treatment must be strictly controlled to avoid the destruction of gut microbiota diversity caused by broad-spectrum antibiotics.

### 3.2 Probiotics

Probiotics, as live microorganisms beneficial to host health, improve the structure and function of the gut microbiota in HF patients by directly supplementing beneficial bacteria, competing for nutrient substrates, or regulating host immunity (Hill et al., 2014). Currently, the most studied strains include *Saccharomyces boulardii*, *Lactobacillus rhamnosus GR-1*, and *Lactobacillus plantarum* has been shown in randomized, double-blind trials to increase LVEF, reduce left atrial diameter, and lower total cholesterol and uric acid levels in HF patients (Costanza et al., 2015). In a model of HF, *Lactobacillus rhamnosus GR-1* can significantly reduce left ventricular hypertrophy and improve left ventricular systolic and diastolic function. Its mechanism is related to increasing the abundance of gut bacteria that produce SCFAs and inhibiting myocardial fibrosis (Gan et al., 2014). Furthermore, *Bifidobacterium BB-12* in probiotic yogurt can reduce the level of soluble tumor necrosis factor and apoptosis-inducing factor (sTWEAK), thereby alleviating the systemic inflammatory state in chronic HF patients (Pourrajab et al., 2022b), providing evidence-based support for the application of probiotics in the adjuvant treatment of HF.

### 3.3 Prebiotics

Prebiotics, as substrates that are not digested and absorbed by the host but can selectively stimulate the growth of beneficial bacteria in the colon, indirectly regulate the structure of the gut microbiota by providing fermentation raw materials for SCFA-producing bacteria. Verhaar et al. pointed out that prebiotics such as fructooligosaccharides and inulin can significantly increase the abundance of Bifidobacteria and *Lactobacillus* in the intestines of HF-related model animals, and increase the levels of SCFAs in feces and serum; while SCFAs can inhibit the NF-κB inflammatory pathway by activating GPR41/43 receptors, while improving intestinal barrier integrity and reducing LPS translocation, thereby alleviating myocardial inflammation and remodeling (Verhaar et al., 2020). Chen et al. (2023) further confirmed the cardioprotective effects of high-fiber diet and SCFAs in mice with isoproterenol (ISO)-induced cardiac hypertrophy. The study showed that both high-fiber diet and acetate supplementation significantly improved physiological parameters, cardiac function and myocardial fibrosis in model mice, and both interventions caused obvious changes in the composition and abundance of gut microbiota. More importantly, through microbiota analysis and bioinformatics integration, Chen et al. clearly proposed that gut microbiota may be a key mediator of the beneficial effects of high-fiber diet and acetate on cardiac hypertrophy, further supporting the mechanism by which prebiotics improve HF via regulating gut microbiota.

### 3.4 Microbial metabolic regulator

These drugs reduce the cardiotoxicity of harmful metabolites (such as TMAO) in the gut microbiota by blocking the synthesis pathway of harmful metabolites in the gut microbiota. 3,3-Dimethyl-1-butanol (DMB) is a typical example, which reduces TMA production by inhibiting the activity of TMA lyase in the gut microbiota, thereby reducing the synthesis of TMAO in the liver. Wang et al. found in a stress overload-induced HF mouse model that DMB intervention significantly reduced serum TMAO levels, alleviated myocardial hypertrophy and fibrosis, and improved left ventricular function; its mechanism was related to the inhibition of TMAO-mediated myocardial oxidative stress and reduction of NF-κB pathway activation (Wang et al., 2020) (see Table 2).

**Table 2:** Modern medical interventions for HF by regulating gut microbiota.

Category	Intervention	Model	Mechanism	Key microbiota/metabolite changes	Cardiac function outcome	References
Antibiotics	polymyxin B, tobramycin (CO)	CHF patients (NYHA class III-IV), *n* = 10, 8 weeks	Selective intestinal decontamination, inhibit the proliferation of Gram-negative bacteria, reduce LPS entry into the blood	Intestinal endotoxin ↓; Monocyte CD14 expression ↓; IL-1/IL-6/TNF-α ↓	Endothelial function improved; Inflammatory markers decreased	Conraads et al. (2004) and Nagatomo and Tang (2015)
	Vancomycin (AE)	Dahl S rats, Myocardial I/R model	Inhibits translocation of Gram-positive bacteria, reduces gut-derived inflammation, and protects intestinal barrier	Gram-positive bacteria ↓; Systemic inflammation ↓	Left ventricular infarct size ↓27%; Cardiac function recovery ↑35%	Lam et al. (2012)
	Rifaximin (RCT)	HFrEF patients (LVEF<40%, NYHA class II-III), *n* = 151, 3 months	Non-absorbable antibacterials, inhibit NF-κB, promote growth of *Lactobacillus*/*Bifidobacterium*, reduce LPS translocation	Some bacteria in class Clostridia ↓; Altered gut microbiota composition with no significant difference in diversity	LVEF showed no significant improvement (vs control, *p* = 0.22); TMAO/CRP remained unchanged	Awoyemi et al. (2021)
Probiotics	Vancomycin + probiotics (GoodBelly containing *Lactobacillus plantarum* 299v + *Bifidobacterium lactis* Bi-07) (AE)	Dahl S rats, Myocardial I/R model	Supplements beneficial bacteria, competitively inhibits pathogenic bacteria, regulates intestinal inflammatory microenvironment	*Lactobacillus* and *Bifidobacterium* ↑; Systemic inflammation ↓	Infarct size ↓29%; Cardiac function recovery ↑23%	Lam et al. (2012)
	*Lactobacillus rhamnosus* GR-1 (AE)	SD rats, LAD ligation-induced CHF model	Promotes growth of SCFA-producing microbiota, inhibits myocardial fibrosis, alleviates inflammatory signaling in the gut-heart axis	Abundance of SCFA-producing bacteria (*Eubacterium*, *Roseburia*, *Ruminococcus*) ↑; SCFAs ↑	LV hypertrophy ↓; LVEF/LVFS significantly improved; Both systolic and diastolic functions improved	Gan et al. (2014)
	Saccharomyces boulardii (RCT)	Chronic systolic HF patients (LVEF < 45%), *n* = 33, 3 months	Regulates intestinal immunity, reduces inflammatory metabolites, improves intestinal barrier integrity	Total cholesterol ↓; Uric acid ↓; hsCRP ↓	LVEF ↑; Left atrial diameter ↓	Costanza et al. (2015)
	Saccharomyces boulardii (RCT)	HFrEF patients (LVEF<40%), *n* = 150, 3 months	Regulates gut microbiota balance, improves immune metabolism	No significant change in microbiota diversity; TMAO unchanged	LVEF showed no significant improvement (vs control, *p* = 0.21); NT-proBNP slightly increased	Mayerhofer et al. (2018)
	probiotic yogurt (RCT)	CHF patients (LVEF<40%), *n* = 90, 10 weeks	Supplements *Bifidobacterium*, regulates immune-gut axis, reduces systemic inflammation	Probiotic yogurt group’s sTWEAK ↑; inflammatory markers improved	Systemic inflammation ↓ (elevated sTWEAK indicates reduced apoptosis induction)	Pourrajab et al. (2022a)
Prebiotics	Inulin/Fructooligosaccharides (AE)	C57BLK/6 mice, HFD-induced Hypertensive model	Provides fermentable substrates for SCFA-producing bacteria, enriches beneficial commensal microbes	*Bifidobacterium* ↑; *Lactobacillus* ↑; fecal/serum SCFAs ↑	Improves intestinal barrier, reduces LPS leakage and myocardial inflammation via GPR41/43/NF-κB pathway	Verhaar et al. (2020)
	High-fiber diet/Acetate supplementation (AE)	C57BLK/6 mice, ISO-induced cardiac hypertrophy model	Dietary fiber is fermented by gut microbiota to produce SCFAs (acetate, butyrate)	SCFA-producing microbiota ↑; acetate ↑; butyrate ↑; altered microbiota diversity	Physiological parameters improved; cardiac function improved; cardiac fibrosis ↓	Chen et al. (2023)
Microbial metabolic regulator	DMB (AE)	C57BL6/J mice, AB/TAC-induced HF model	Inhibits the activity of gut microbiota TMA lyase (CutC/D), reduces the conversion of TMA to TMAO	Plasma TMAO ↓; TMA-producing bacteria ↓; butyrate-producing bacteria relatively ↑	LVEF ↑; myocardial hypertrophy ↓; fibrosis ↓ (via TGF-β1/Smad3 ↓, NF-κB ↓)	Wang et al. (2020)
	DMB (AE)	SD rats, LAD ligation-induced CHF model	Inhibits TMA synthesis and reduces circulating TMAO levels	Plasma TMAO ↓; IL-8 ↓	Delayed HF progression; improved cardiac function (via inhibition of IL-8-mediated inflammation)	Li et al. (2019b)
	iodomethylcholine (AE)	C57BLK/6 J mice, TAC-induced CHF model	Irreversibly inhibits CutC/D (TMA lyase), exerts no bactericidal effect, and slightly modulates microbiota structure	Plasma TMAO significantly ↓ (>95%); BNP ↓; pro-fibrotic genes ↓	Cardiac remodeling ↓; cardiac function improved; renal function protected	Organ et al. (2020)

AE, Animal experiment; CO, Clinical observation; RCT, Randomized controlled trial; NT-proBNP, N-terminal pro-brain natriuretic peptide; TAC/AB, Transverse aortic constriction/Aortic banding; LAD, Left anterior descending coronary artery; I/R, ischemia–reperfusion.↑ = increased/improved, ↓ = decreased/reduced.

Western medicine has shown therapeutic potential in regulating the gut microbiota and improving enterogenic inflammation and metabolic disorders in HF patients through antibiotic regimens are mostly limited to small sample and short-term follow-up stages, and whether long-term probiotic supplementation leads to excessive microbiota proliferation and the long-term effects of DMB use on liver metabolism have not been fully verified. Secondly, antibiotics present a trade-off between short-term decontamination and long-term gut microbiota disruption. Once interventions such as SID and probiotics are discontinued, the gut microbiota quickly reverts to its baseline disordered state. Long-term antibiotic use, however, carries the risk of drug resistance and fungal translocation, leading to a cyclical dilemma of treatment-relapse-retreatment (Costanza et al., 2015; Awoyemi et al., 2021). Probiotic, prebiotic, or antibiotic regimens mostly stay in small samples and short-term follow-up periods. It has not been fully verified whether long-term probiotic supplementation leads to overproliferation of flora and how long-term use of DMB affects liver metabolism. Second, there is a trade-off between the short-term decontamination provided by antibiotics and the long-term destruction of flora. Once SID, probiotics, and other interventions are discontinued, the flora quickly returns to the baseline disorder state; while long-term use of antibiotics faces the risk of drug resistance and fungal migration, resulting in a cyclical dilemma of treatment-relapse-retreatment (Ponziani et al., 2017). Finally, mechanistic studies are insufficient; most studies only observe changes in correlation, lacking molecular pathway analysis, and there is no unified standard for the relationship between drug dosage and effect, making it difficult to replicate and generalize the results. These limitations collectively prevent such drugs from becoming a standard treatment for HF. Previous studies have suggested that conventional Western medicines may even aggravate gut microbiota dysbiosis in some cases, thus weakening the protective function of the gut-heart axis. This therapeutic gap suggests that relying solely on Western medicines cannot fully address the complex problem of gut microbiota dysbiosis in HF. The combined therapeutic model integrating complementary advantages of traditional Chinese and Western medicine may achieve better efficacy via the synergistic effects of overall regulation of microbiota ecology and precise intervention on key metabolic pathways, providing a valuable research direction for gut-heart axis-targeted therapy of HF.

## 4 Treatment of HF with TCM based on gut microbiota

### 4.1 Regulating microbiome fermentation potential and intestinal barrier

**Figure 3:**
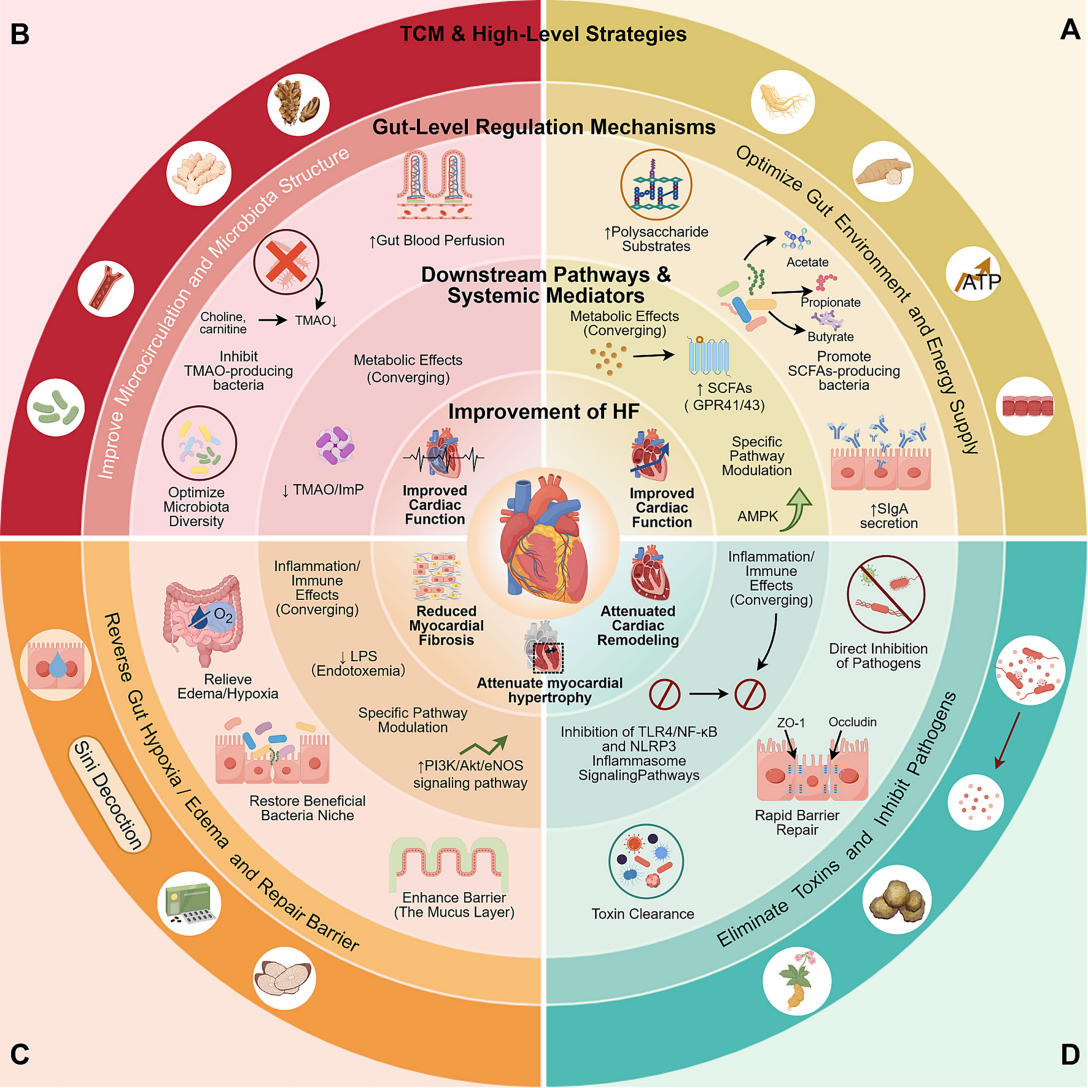
TCM regulates the gut microbiota to improve HF (by Figdraw).

In the pathophysiology of HF, for the situation of insufficient intestinal fermentation and energy metabolism imbalance, single Chinese medicines, represented by Astragalus membranaceus and Ginseng, have core active ingredients that act as highly efficient prebiotic substrates in the gut. Animal studies have shown that Astragalus polysaccharides (APS) can significantly increase the abundance of *Bifidobacterium* and *Lactobacillus*, reduce the ratio of Bacteroidetes and Proteobacteria, and enhance intestinal barrier function and inhibit systemic inflammation by promoting the proliferation of SCFAs-producing bacteria such as Ruminococcus (Jia et al., 2020; Xu et al., 2018; Zhang et al., 2012). These gut-level regulations ultimately improve cardiac function and inhibit cardiac remodeling by activating the AMPK pathway and inhibiting signaling pathways such as TLR-4/NF-κB (Tuo et al., 2021; Liu et al., 2019). The polysaccharides and saponins in ginseng and its processed products (such as red ginseng and black ginseng) can specifically stimulate the proliferation of *Lactobacillus* and *Bacteroides*. In particular, black ginseng has been shown to increase the abundance of *Lactobacillus*, promote the production of acetic acid and butyric acid, and reduce myocardial inflammation by activating the vagus nerve pathway (Zhou et al., 2016; Jia et al., 2020; Dou et al., 2024). In addition, high molecular weight polysaccharides, gypenosides, rhodioloside, and licorice extracts from Cordyceps sinensis have all shown the potential to enrich beneficial bacteria (such as *Parabacteroides goldsteinii* and *Akkermansia*), inhibit pathogenic bacteria, enhance the intestinal barrier, and reduce endotoxin entry into the bloodstream. Specific mechanisms are detailed in Table 3 (Wu et al., 2019b; Chen L. et al., 2016; Yuan et al., 2019; Shi G. et al., 2022). The total polysaccharides in the TCM compound Sijunzi Decoction can synergistically upregulate intestinal secretory IgA, strengthen the mechanical barrier, reduce circulating endotoxins, and thus inhibit the myocardial TLR4/NF-κB pathway, improving cardiac function in HF rats (Lu et al., 2021; Wu et al., 2014). Shengmai San and related preparations (such as Shenmai injection) focus on regulating the co-metabolism of microorganisms and the host, and repairing metabolic disorders caused by microbial imbalance (Ming et al., 2022a; Li L. et al., 2025b). In addition, compound formulas such as Buyang Huanwu Decoction and Qishen Granules also exert anti-HF effects by improving gut microbiota diversity, repairing the intestinal barrier, and reducing TMAO levels (Table 3; Figure 3A).

**Table 3:** TCM and compound formulas for the treatment of HF by regulating gut microbiota.

Herb/formula name	Active ingredients	Model	Mechanism of action	Associated microbiota changes	References
Astragalus (Huangqi)	Astragaloside IV, Astragalus polysaccharides, Astragalus decoction (AMD)	C57BL-6 mice, ISO -induced HF	Inhibits inflammatory cytokine expression, attenuates intestinal mucosal injury; reverses gut dysbiosis; promotes SCFA generation, enhances intestinal barrier function	Bacteroidetes*↑,* Lachnospiraceae*↑, Blautia* spp.*↑, Clostridium* spp.*↑. Ruminococcus↑;* Firmicutes*↓,* Proteobacteria*↓*	Cui et al. (2018) and Du et al. (2022)
Ginseng (Renshen)	Processed products: Red Ginseng, Black Ginseng; Ginsenoside Rb1	DUSP-1/VDAC1 transgenic or knockout mice, TAC-induced HF; SD rats, ISO-induced HF	Improves intestinal metabolic environment, facilitates ginsenoside absorption; regulates gut microbiota; promotes SCFAs production, activates vagus nerve pathway	Lachnospiraceae*↑,* Muribaculaceae *↑;* Red Ginseng: Lactic acid bacteria *↑, Escherichia coli ↓* Black Ginseng: Lactobacillales *↑,* Erysipelotrichales *↓*	Pu et al. (2024), Dou et al. (2024), and Li L. et al. (2025b)
Cordyceps (Dongchongxiacao)	Adenosine, Cordycepin, High molecular weight polysaccharides (H1 fraction), Natural Cordyceps sinensis polysaccharides (NCSP)	C57BL/6 mice, DOX-induced HF; D-galactose-induced HF; HFD-induced gut dysbios model	Reverses gut dysbiosis; reduces intestinal permeability, alleviates endotoxemia; inhibits release of intestinal and systemic inflammatory cytokines; enhances intestinal immune function, regulates Th17/Treg balance	*Parabacteroides goldsteinii*↑; *Escherichia coli*↓, *Shewanella algae*↓; *Bifidobacterium*↑, *Lactobacillus*↑	Wu et al. (2019a), Li Y. et al. (2024), and Liu S. Y. et al. (2025)
Gynostemma (Jiaogulan)	Gypenosides (GpS)	Wistar rats, ISO-induced HF	Inhibit inflammation and pro-inflammatory cytokines; inhibit cardiomyocyte apoptosis and reduce caspase-3, caspase-6, caspase-9 expression; modulate gut microbiota, ameliorate intestinal inflammatory microenvironment, and enhance intestinal barrier integrity	*Lactobacillus*↑, *Allobaculum*↑ Firmicutes↑; Bacteroidetes↓, *Desulfovibrio*↓, *Prevotella*↓	Zhang X. et al. (2023)
Rhodiola (Hongjingtian)	Salidroside (SAL)	C57BL/6 mice, Furan-induced liver injury model^†^	Reduce pro-inflammatory cytokine levels, decrease MDA content, and enhance antioxidant enzyme activity; improve gut microbiota diversity, reduce endotoxin production, and preserve intestinal barrier integrity	*Akkermansia*↑; Proteobacteria↓	Yuan et al. (2019)
Licorice (Gancao)	Licorice water extract (LWE)	C57BL/6 mice, DSS-induced colitis model^†^	Inhibit the NF-κB signaling pathway, modulate gut microbiota, and enhance intestinal barrier function	*Bacteroides↑, Lactobacillus↑*; *Escherichia*-*Shigella↓, Turicibacter↓*	Shi G. et al. (2022)
Sijunzi Decoction	Total polysaccharides of Si Jun Zi Tang	SD rats, LAD-induced HF	Enrich probiotics, enhance intestinal mucosal immunity (SIgA↑), and block the linkage between gut dysbiosis and myocardial inflammation	Bacteroidetes↑, *Lactobacillus*↑; Firmicutes↓, Muribaculaceae↓	Lu et al. (2021)
Buyang Huanwu Decoction	-	SD rats, AAC-induced HF	Delay abnormal gut microbiota remodeling, enhance intestinal tight junction protein expression, and reduce serum TMAO levels	Bacteroidetes↑; Firmicutes↓, Proteobacteria↓, Verrucomicrobia↓	Weng et al. (2023)
Shengmaiyin	Ginsenosides; Lignans; Ophiopogonin and active polysaccharides	SD rats, Cardiac hypertrophy-induced HF	Regulate the imbalance of gut microbiota composition, ameliorate gut microbial metabolic disorders; modulate microbe-host co-metabolism and repair intestinal barrier function	Bacillota↑, Bacilli↑, Lactobacillales↑, Lachnospiraceae↑; Bacteroidetes↓, Actinobacteria↓, Erysipelotrichia↓, Saccharomonadia↓	Ming et al. (2022b)
Shenmai Injection	-	SD rats, AAC-induced HF	Restore gut microbiota homeostasis, modulate microbe-host co-metabolism, and repair intestinal barrier integrity	*Lactobacillus*↑, *Bifidobacterium*↑; Proteobacteria↓, *Enterococcus*↓	Li L. et al. (2025b)
Qishen Granules	-	SD rats, LAD-induced HF	Reduce plasma LPS levels and improve intestinal barrier protective function	*Bacteroides*↑, *Lactobacillus*↑, *Akkermansia*↑; Proteobacteria↓, *Desulfovibrio*↓	Gao K. et al. (2023)
*Salvia miltiorrhiza* (Danshen)	Tanshinone IIA, Salvianolic acids	SD rats, ISO-induced HF model; LAD-induced HF	Improve lipid and amino acid metabolism; regulate intestinal SCFAs and BAs; alleviate myocardial pathological injury; improve metabolic disorders	Bacteroidetes↑, *Lactobacillus*↑, *Akkermansia* ↑; Firmicutes↓, *Romboutsia*↓	Cai et al. (2024), Zhu et al. (2024)
Turmeric (Jianghuang)	Curcumin	LDLR^−^/^−^ mice, Western diet-induced atherosclerosis and glucose intolerance model (indirectly associated with HF)	Reshape gut microbiota, reduce intestinal permeability, and inhibit the LPS-mediated myocardial inflammatory pathway	*Lactobacillus*↑, *Bifidobacterium*↑, Bacteroidetes↑; Clostridiales↓	Ghosh et al. (2014) and Muttiah and Hanafiah (2025)
Safflower (Honghua)	Hydroxysafflor yellow A (HSYA)	C57BL/6 mice, LAD ligation-induced HF	Promote the growth of SCFA-producing bacteria, enhance intestinal integrity, and improve microcirculation	*Butyricimonas*↑, *Prevotella gracilis* ↑	Mao et al. (2025), Liu et al., 2018
Fuzi Decoction	Alkaloids; saponins; flavonoids	SD rats, LAD -induced CHF	Regulate gut microbiota metabolism, increase SCFA levels, and improve the valine-leucine-isoleucine biosynthesis pathway	Lachnospiraceae↑, *Bacteroides*↑, Blautia↑; Streptococcaceae↓, Enterococcaceae↓	Gao T. et al. (2023)
Sini Decoction	-	Wistar rats, LAD-induced HF	Restore intestinal mucosal integrity, inhibit the myocardial TLR4/NF-κB pathway, and improve myocardial structural disorder	Bacteroidetes*↑,* Spirochaetae*↑*; Proteobacteria*↓*	Zhao et al. (2022b)
Shenfu Injection	-	SD rats, DOX-induced CHF	Thicken the intestinal mucus layer, reduce LPS translocation into the blood, and activate the PI3K/Akt/eNOS cardioprotective pathway	*Lactobacillus↑, Bacteroides↑*; Proteobacteria*↓, Desulfovibrio↓*	Zhao et al. (2022a) and Zhao Z. et al. (2025)
Baoyuan Decoction	-	SD rats, ISO-induced CHF	Improve myocardial hypertrophy; reverse fecal metabolism disorders in model rats; inhibit arginine and tryptophan derivatives and their downstream pro-hypertrophy, pro-inflammation and pro-oxidation pathways	Bacteroidetes↑; Firmicutes↓	Du Z. et al. (2020)
Qili Qiangxin Capsules	-	SD rats, TAC-induced HF	Regulate the microbiota-inflammation axis, inhibit the NLRP3 inflammasome, reduce TMAO levels, and decrease myocardial fibrosis	*Phascolarctobacterium↑, Intestinimonas↑, Bacteroides↑; Prevotella↓, Alloprevotella↓*	Lu et al. (2022) and Zhu et al. (2025)
Xiao Qing Long Tang	-	Dahl salt-sensitive rats, High-salt diet-induced HFpEF	Increase SCFA production, repair intestinal barrier; inhibit myocardial inflammation and fibrosis	*Lactobacillus↑*; Proteobacteria*↓*	Zhou et al. (2019b)
Rhubarb (Dahuang)	Emodin	ICR mice, DOX-induced HF	Remodel gut microbiota structure; promote production of beneficial gut metabolite IPA; inhibit ferroptosis; improve heart function	*Bifidobacterium↑, Lactobacillus↑*; Verrucomicrobiota*↓, Desulfovibrio↓*	Hu et al. (2024b)
Coptis (Huanglian)	Berberine	ApoE^−^/^−^ mice, HFD-induced atherosclerosis (precursor lesion associated with HF)	Inhibit pathogenic bacteria proliferation, promote SCFA-producing bacteria growth; repair intestinal barrier and inhibit inflammatory pathways	*Lactobacillus*↑, *Bifidobacterium*↑, *Clostridium*↑, *Roseburia*↑, *Akkermansia*↑; *Enterobacter*↓, *Enterococcus*↓, TMAO-producing bacteria↓	Cui H. et al. (2023) and Zhu et al. (2018)
Purslane (Machixian)	*Portulaca oleracea* polysaccharides	Kunming mice, DSS-induced ulcerative colitis^†^	Increase the anti-inflammatory cytokine IL-10, decrease the pro-inflammatory cytokines TNF-α and IL-6, and rectify microecological imbalance	*Bifidobacterium*↑, *Lactobacillus*↑; *Enterobacter*↓, *Enterococcus*↓	Feng et al. (2015)
Giant Knotweed (Huzhang)	Resveratrol (RSV)	ApoE^−^/^−^ mice, choline diet-induced atherosclerosis; SD rats, AAC-induced HF	Reshape gut microbiota structure, inhibit the NF-κB pathway, and alleviate myocardial inflammation and fibrosis	Bacteroidetes↑, *Lactobacillus*↑; Firmicutes↓	Chen et al. (2016b) and Li and Ma (2021)
Si-Miao-Yong-An Decoction	-	SD rats, Myocardial ischemia–reperfusion injury HF	Enhance intestinal barrier function, reduce LPS translocation into the blood, and inhibit the activation of the TLR4/NF-κB pathway	Bacteroidetes↑, *Alloprevotella*↑; Prevotellaceae_Ga6A1↓, Prevotellaceae_NK3B3↓	Cui Y. et al. (2023)
Qige Huxin Formula	-	C57BL/6 mice, ISO -induced HF	Reverse gut microbiota dysbiosis, upregulate colonic ZO-1 and occludin expression, reduce intestinal permeability, and decrease LPS translocation into the blood	Bacteroidetes*↑,* Muribaculaceae*↑,* Lachnospiraceae*↑*; Firmicutes*↓,* Staphylococcaceae*↓,* Corynebacteriaceae*↓*	Shi L. et al. (2022)
Xiexin Tang	-	SD rats, HFD-induced obesity^†^ (Metabolic-associated HF)	Upregulate the activity of key SCFA synthesis enzymes (ACK, MMD) and enhance the SCFA-producing capacity of gut microbiota	Bacteroidetes*↑;* Firmicutes*↓*	Xiao et al. (2019)

AAC = Abdominal Aortic Coarctation; DSS=Dextran sulfate sodium; HFD=High-fat diet. “↑” denotes increased levels; “↓” denotes reduced levels; “-” indicates that the article did not mention. Entries marked with † indicate studies using indirect models (not direct HF models); their relevance to HF is inferred from shared pathological mechanisms (intestinal barrier injury, gut dysbiosis) and requires further validation in HF-specific models.

### 4.2 Reshaping the oxygen gradient and improving intestinal microecology

HF patients commonly have intestinal “congestion,” which is essentially tissue hypoxia and intestinal ecological remodeling caused by mesenteric microcirculation disorders. TCM for promoting blood circulation and removing blood stasis can indirectly exert cardioprotective effects by improving microcirculation, restoring intestinal blood perfusion, optimizing the microbial living environment, correcting the intestinal oxygen gradient (Figure 3B). Early studies have confirmed that *Salvia miltiorrhiza* extract can significantly increase mesenteric circulating blood flow, enhance the oxygen tolerance of intestinal mucosal epithelial cells, and lay the foundation for stable colonization of the microbial community (Liu et al., 2009). Active ingredients, represented by ginseng ketone IIA, have been shown in animal models to attenuate the extreme hypoxic microenvironment caused by blood stasis by improving intestinal mucosal blood perfusion, thereby regulating the gut microbiota structure, regulating SCFAs production, and improving HF-related pathological damage by mediating the gut microbiota-inflammatory axis to inhibit the activation of the TLR4/NF-κB and NLRP3 inflammasome pathways (Hua et al., 2025). Curcumin can significantly remodel the composition of the gut microbiota, increasing beneficial bacteria such as *Lactobacillus* and *Bifidobacterium*. Simultaneously, it reduces intestinal permeability and decreases LPS entry into the bloodstream by upregulating the expression of tight junction proteins in the intestinal epithelium (Yu et al., 2019; Ghosh et al., 2014). Injectable safflower yellow pigments (such as hydroxysafflower yellow pigment A, HSYA) have also been preliminarily shown to increase the abundance of SCFAs-producing bacteria, enhance intestinal integrity, and reverse gut microbiota imbalance (Liu et al., 2018). However, more HF-specific evidence is needed (Table 3).

### 4.3 Reversing the hypoxic microenvironment and improving microcirculation

In the later stages of HF, the retention of dampness and severe intestinal edema caused by cardiac and renal failure create an extreme hypoxic environment and barrier function collapse in the intestine, leading to severe dysbiosis and endotoxemia. Improving microcirculation creates the necessary conditions for the colonization of obligate anaerobic probiotics, thereby blocking LPS translocation caused by barrier collapse (Figure 3C). Si Ni Tang (*Aconitum carmichaelii*, *Zingiber officinale*, Glycyrrhiza uralensis) significantly improved intestinal mucosal flora disorder, restored intestinal mucosal integrity, and reduced serum endotoxin levels in an adriamycin-induced HF rat model. Its mechanism involves reducing the entry of pro-inflammatory factors such as enterogenic TNF-*α* and IL-1β into the bloodstream, inhibiting the activation of the myocardial TLR4/NF-κB pathway, and ultimately improving LVEF (Zhao et al., 2022b). The mechanism of action of Qili Qiangxin Capsules (containing *Aconitum carmichaelii*, Astragalus membranaceus, etc.) has been verified through both animal experiments and clinical studies. In aortic coarctation HF model, this compound can restore the abundance of beneficial bacteria, increase SCFA-producing bacteria, reduce LPS production by regulating the flora, specifically inhibit the activation of NLRP3 inflammasomes in the myocardium and intestines, and reduce myocardial fibrosis (Lu et al., 2022; Wang et al., 2022). A randomized clinical study further showed that Qili Qiangxin Capsules can reduce systemic inflammation by lowering serum TMAO levels and repairing the intestinal barrier in patients with HF, thereby improving cardiac function (Zhu et al., 2025). For acute exacerbations of HF, Shenfu injection can reduce LPS entry into the bloodstream by increasing the thickness of the intestinal mucus layer, while simultaneously activating the myocardial PI3K/Akt/eNOS protective pathway (Zhao et al., 2022a; Zhao Z. et al., 2025). In addition, compound formulas such as Xiao Qinglong Decoction, Baoyuan Decoction, and Fuzi Decoction also improve HF through common mechanisms such as increasing SCFAs levels, reducing the abundance of pro-inflammatory bacteria, and repairing the intestinal barrier (Table 3) (Li et al., 2020; Zhou et al., 2019a; Du Z. et al., 2020; Wu et al., 2022; Gao T. et al., 2023).

### 4.4 Clearing metabolic toxins and blocking inflammatory storms

During the progression of HF, pathologically, severe damage to the intestinal barrier and the collapse of the microbiota structure lead to endotoxemia and cytokine storms (common in the acute exacerbation phase of HF). Herbs with heat-clearing and purgative properties are characterized by rapid action and strong targeting. They can directly target and inhibit the proliferation of pathogenic bacteria, clear enterogenic toxins, rapidly repair the intestinal barrier, and effectively inhibit key inflammatory pathways to block the vicious cycle of the gut-heart axis (Figure 3D). Berberine, the core active ingredient of Coptis chinensis, is a representative substance for regulating the gut microbiota. Preclinical studies suggest that berberine may remodel gut microbiota composition by inhibiting the HF-related pathogenic bacteria while strongly promoting the abundance of SCFA-producing bacteria, thus cutting off the endotoxin generation pathway at its source (Cui H. et al., 2023; Zhang et al., 2012; Wang et al., 2017). It is noteworthy that berberine possesses intestinal specificity, enabling it to efficiently repair the intestinal barrier, upregulate ZO-1 and occludin expression, and significantly reduce serum LPS and pro-inflammatory factor levels (Cui H. et al., 2023; Zhu et al., 2018). Furthermore, there is evidence suggesting that berberine has the potential to reduce the abundance of TMAO-producing bacteria (Zhang C. et al., 2025). The active components of rhubarb, such as rhein and rhein anthraquinone glycosides, focus on repairing intestinal structure and optimizing gut microbiota composition. Rhein can restore the expression and distribution of ZO-1 in intestinal epithelium and directly weaken NF-κB activation (He et al., 2019). Rhubarb anthraquinone glycosides preparation can increase the abundance of mucus-degrading bacteria and SCFAs-producing bacteria, optimize the Firmicutes/Bacteroidetes ratio (F/B ratio), and thus enhance intestinal barrier function (Zhang H. Y. et al., 2021). Resveratrol in *Polygonum cuspidatum* extract has both antioxidant and anti-inflammatory effects. It can specifically reduce the abundance of TMAO-producing bacteria by remodeling the gut microbiota structure, thereby lowering plasma TMAO levels and inhibiting myocardial fibrosis (Chen M. L. et al., 2016; Li and Ma, 2021). At the compound level, Simiao Yong’an Decoction enhances intestinal barrier function, reduces LPS entry into the bloodstream, and thereby inhibits TLR4/NF-κB pathway activation, reducing the incidence of HFafter myocardial ischemia–reperfusion injury (Cui H. et al., 2023) (Table 3).

Although the evidence reviewed above provides preliminary support for the therapeutic potential of TCM in improving HF through gut microbiota modulation, several challenges remain before clinical translation can be achieved. The foremost difficulty lies in attributing therapeutic effects to specific active ingredients. Herbal formulas typically comprise dozens of chemical constituents with potential synergistic or antagonistic effects, making it difficult to attribute cardioprotective efficacy to specific active compounds acting through defined microbial targets. Most clinical trials evaluate the overall effect of complete formulas, and dismantling studies targeting microbiota-mediated mechanisms remain scarce (Zhou et al., 2025). Closely related to this challenge is the pharmacokinetic uncertainty of TCM compounds. Many active molecules in TCM—particularly polysaccharides and saponins—exhibit low oral bioavailability and require extensive biotransformation by gut microbiota before exerting pharmacological activity. For instance, ginsenoside Rb1 must be hydrolyzed by intestinal bacteria to compound K for significant bioactivity, a process highly dependent on individual microbial composition (Lin et al., 2021). This microbiota-dependent pharmacokinetics implies that the same formula may produce variable metabolite profiles across patients, increasing inter-individual heterogeneity in clinical outcomes. Furthermore, unlike targeted interventions such as probiotics or DMB, TCM exerts broad-spectrum modulation of gut microbiota, simultaneously altering the relative abundance of multiple genera. Establishing causal links between specific microbial changes and cardiac protection still relies on germ-free animal models or fecal microbiota transplantation experiments (Zhang et al., 2022). At the clinical level, the existing evidence is predominantly derived from animal studies, with few well-designed human clinical trials; those available generally have small sample sizes, short follow-up periods, and inconsistent microbiota detection methodologies (Cui H. et al., 2023). Variability in herbal sourcing, processing, and preparation further affects the stability and reproducibility of active components.

In summary, neither conventional Western nor TCM approaches alone can fully address the complex pathological interplay of gut-heart axis dysregulation during HF progression. Integrating the precision-targeted interventions of Western medicine with the holistic regulatory advantages of TCM through a synergistic treatment strategy may achieve complementary strengths, offering a more clinically translatable approach for microbiota-targeted HF therapy.

## 5 The efficacy of TCM decoction on the treatment of HF combined with modern medicine from the perspective of the “gut-heart” axis

### 5.1 Differences in intestinal microbiota characteristics of HF clinical phenotype

#### 5.1.1 Characteristics of gut microbiota of different HF subtypes

Multiple studies have revealed that different HF subtypes exhibit both common and specific dysregulation patterns in gut microbiota structure and metabolic function (Table 4). Overall, all HF subtypes showed a trend of decreased *α* diversity, but the degree of decrease differed: the decrease in gut microbiota diversity was most significant in HFrEF patients, while it was relatively mild in HFpEF (Luedde et al., 2017; Beale et al., 2021).

**Table 4:** Characteristics of gut microbiota of different HF subtypes.

HF subtypes	α-Diversity	Core phylum-level alterations	Key differential genera (vs. healthy controls)	Microbial metabolites	Underlying mechanisms	Research evidence
HFrEF	Significantly decreased (30–45% lower than healthy controls, *p* < 0.01); the decrease was more significant in severe patients (NYHA class III-IV, over 40% lower than healthy controls)	1. Relative abundance of Firmicutes decreased; 2. Relative abundance of Bacteroidetes decreased slightly; 3. Relative abundance of Proteobacteria increased; 4. Abundance of Actinobacteria decreased	Reduction of beneficial bacteria: *Prevotella, Propionibacterium freudenreichi,* butyrate-producing bacteria *Bifidobacterium, Lactobacillus* Enrichment of pro-inflammatory bacteria: *Candida, Campylobacter, Shigella, Salmonella, Yersinia, Escherichia coli,* Enterobacteriaceae	1. Decreased: SCFAs (butyrate, acetate, propionate) 2. Increased: TMAO, LPS, phenylacetylglutamine (PAGln)	1. Reduced cardiac output causes intestinal hypoperfusion, and impaired venous return induces intestinaledema, forming a hypoxic microenvironment; 2. Pro-inflammatory cytokines (lL-6, TNF-a) and ROS damage the intestinal epithelialbarrier and disrupt gut microbiota homeostasis; 3. Gut microbiota dysbiosis in severe patients exacerbates systemic inflammatory responses, furtherdeteriorating cardiac function.	Luedde et al. (2017), Kummen et al. (2018), Kamo et al. (2017), Mirhosseini et al. (2025), Pasini et al. (2016), Katsimichas et al. (2018), and Sun et al. (2021)
HFpEF	Mildly decreased (18% lower than healthy controls, *p* < 0.05); the reduction amplitude was more moderate than that in HFrEF patients	1. The F/B ratio decreased by 30%; 2. The relative abundance of Proteobacteria increased slightly	Reduction of beneficial bacteria: *Ruminococcus, Butyricicoccus, Sutterella, Lachnospira,* Ruminococcaceae (ruminants-related taxa)*, Ruminiclostridium* Enrichment of pro-inflammatory bacteria: Erysipelotrichaceae*, Lactobacillus, Enterococcus*	1. Decreased: IPA, SCFAs (acetate, propionate, butyrate), secondary bile acids 2. Increased: TMAO, indoxyl sulfate	1. The gut microbiota-barrier-inflammation network drives cardiac pathological injury, characterized by areduction in anti-inflammatory bacteria and enrichment of pro-inflammatory bacteria; 2. Decreases in SCFAs and lPA weaken myocardial protection.	Shah et al. (2020), Beale et al. (2021), Bassis et al. (2019), Drapkina et al. (2022), Guivala et al. (2024), Luo et al. (2025), Schiattarella et al. (2019), Vacca and Schiattarella (2024), Palm et al. (2025), and Mamic et al. (2021)
HFmrEF	Moderately decreased (25–30% lower than healthy controls, *p* < 0.01)	Limited data available; preliminary evidence suggests a similar trend toward HFpEF	Reduction of beneficial bacteria: SCFA-producing related genera Enrichment of pro-inflammatory bacteria: *Enterobacter, Escherichia*-*Shigella*	Decreased: SCFAs (acetate + propionate + butyrate) were generally reduced; TMAO was slightly increased	Inflammation, and disruption of metabolic function Increased conditional pathogens correlate with deterioration of nutritional indicators, exacerbation of intestinal	Palm et al. (2025) and Yang et al. (2024)

In terms of gut microbiota structure, the dysregulation pattern in HFrEF is mainly driven by hemodynamic disturbances. Due to severe intestinal ischemia and hypoxia caused by a significant reduction in cardiac output, beneficial bacteria (especially butyrate-producing bacteria) are significantly reduced in the gut of HFrEF patients, while pro-inflammatory Proteobacteria and various opportunistic pathogens (such as *Shigella* and *Salmonella*) are significantly enriched (Kummen et al., 2018; Pasini et al., 2016). In contrast, the dysbiosis pattern in HFpEF is more focused on metabolic and inflammatory drivers. It is characterized by a consistently decreasing F/B ratio, a particularly pronounced decrease in the abundance of anti-inflammatory species (such as *Ruminococcus*, *Butyricococcus*), and an increase in the abundance of pro-inflammatory bacteria (such as *Erysipelataceae*) (Beale et al., 2021; Bassis et al., 2019; Drapkina et al., 2022). This is consistent with the pathological characteristics of HFpEF often associated with metabolic diseases (Guivala et al., 2024; Luo et al., 2025). The gut microbiota characteristics of heart failure with mid-range ejection fraction (HFmrEF), on the other hand, exhibit a transitional state. Studies suggest a similar metabolic dysregulation pattern to HFpEF, but in terms of gut microbiota composition, it is characterized by a significant increase in opportunistic pathogens such as *Enterobacter* spp. and *Escherichia coli*-*Shigella* spp., which may be related to its nutritional status and intestinal inflammation level (Palm et al., 2025; Yang et al., 2024).

At the metabolite level, all subtypes showed a trend of decreased SCFAs and increased TMAO. However, HFpEF patients also exhibited characteristic decreased levels of the protective metabolite IPA and increased levels of indole sulfate, a tryptophan metabolic byproduct, further highlighting the specificity of their metabolic disorder (Schiattarella et al., 2019; Mamic et al., 2021).

#### 5.1.2 Dynamic changes of microbiota with different NYHA grades

The gut microbiota dysbiosis in HF exhibits stage-specific characteristics, with changes in its composition highly synchronized with NYHA functional classification. This dynamic change is characterized by a gradual decrease in microbiota diversity, a decline in the abundance of beneficial bacteria, and an increase in the abundance of pro-inflammatory/pathogenic bacteria as the NYHA classification increases (Florek et al., 2025). See Table 5 for details.

**Table 5:** Characteristics of gut microbiota with different NYHA grades.

NYHA classes	α-diversity	Core phylum-level alterations	Key differential genera (vs. healthy controls/lower NYHA functional classes)	Microbial metabolites	Underlying mechanisms	Research evidence
NYHA l (mild)	A decreasing trend may have emerged, but the difference from healthy controls may not have reached statistical significance; the downward trend was more pronounced in non-ischemic HFrEF patients	A preliminary downward trend in the F/B ratio was observed; Actinobacteria decreased slightly	Beneficial genera: *Lactobacillus* and *Coprococcus* may decrease (less than 15%); Potential conditional pathogens: Enterobacteriaceae and *Collinsella* may increase (less than 20%)	TMAO levels may have started to increase, and LPS levels may have increased mildly (direct evidence is limited, as most studies have focused on patients with NYHA II-IV)	Cardiac output decreased mildly; in the early stage of intestinal hypoperfusion, the gut microbiota colonization microenvironment changed, but no significant barrier damage was induced	Florek et al. (2025), Pasini et al. (2016), Zhang Z. et al. (2023), and Katsimichas et al. (2018)
NYHA II (mild-to-moderate)	Mildly decreased (18% lower than healthy controls, *p* < 0.05); the reduction amplitude reached 22% in non-ischemic HFrEF patients	Firmicutes decreased slightly and Bacteroidetes increased slightly (no statistical significance); Proteobacteria increased mildly (+12%)	Beneficial bacteria: *Faecalibacterium prausnitzii* and *Blautia* decreased mildly (20–25%); Pathogenic bacteria: *Escherichia*-*Shigella* and *Enterobacter* increased mildly (25–30%)	SCFAs (acetate, propionate) decreased mildly, while TMAO increased mildly	Intestinal ischem ia aggravated; mild impairment of intestinal barrier tight junctions, mild gut microbiotatranslocation, and initial enrichment of pro-inflammatory bacteria occurred	Luedde et al. (2017) and Huang et al. (2022)
NYHA III (moderate)	Significantly decreased (35% lower than healthy controls, *p* < 0.01)	Firmicutes accounted for 48.8% (60.5% in healthy controls); Bacteroidetes increased to 32% (25% in healthy controls); Proteobacteria increased to 18%; Actinobacteria decreased to 8% (12% in healthy controls)	Dominant bacteria: *Escherichia* (pro-inflammatory) and *Bifidobacterium* (anti-inflammatory) showed increased abundance; Deficient bacteria: SCFAs-producing *Agathobacter* was significantly lower than that in healthy controls	SCFAs (butyrate) decreased moderately, LPS increased mildly, and bile acid metabolism was mildly dysregulated (secondary/primary BA ratio increased)	Intestinal wall edema and collagen deposition caused compression of gut microbiota colonization space, excessive proliferation of pro-inflammatory bacteria, and intestinal metabolic dysfunction	Zhang Z. et al. (2023), Florek et al. (2025), Sun et al. (2021), Katsimichas et al. (2018), and Mayerhofer et al. (2017)
NYHA IV (severe)	Extremely decreased (the lowest among all HF stages, 52% lower than healthy controls, *p* < 0.001)	Firmicutes accounted for 51.1% (slightly higher than that in NYHA Class ll, but with lower diversity); Proteobacteria increased significantly to 25%; Bacteroidetes decreased to 20%; Actinobacteria decreased to 5%	Dominant bacteria: *Klebsiella* (pathogenic, positively correlated with infection risk) and *Lactobacillus* (antiinflammatory, but abundance lower than that in NYHA ll) showed increased abundance; Deficient bacteria: *Faecalibacterium prausnitzii* and *Ruminococcus* (SCFAs-producing bacteria) were almost undetectable	SCFAs (acetate, propionate, butyrate) decreased extremely; TMAO increased significantly (positively correlated with HF hospitalization risk); LPS increased significantly	Ischemic necrosis of intestinal mucosal villi led to the marked attenuation of oxygen partial pressure gradient, complete imbalance of gut microbiota niche, and activation of systemic inflammation	Organ et al. (2016), Katsimichas et al. (2018), Sun et al. (2021), and Mayerhofer et al. (2017)

### 5.2 HF treatment integrating TCM and modern medicine based on gut microbiota

#### 5.2.1 Synergistic effect mechanism

Although there are relatively limited studies directly focusing on the regulation of gut microbiota in the treatment of HF with integrated traditional Chinese and Western medicine, there is evidence that gut microbiota is a key link between the two synergistic effects. The synergistic logic of integrated traditional Chinese and Western medicine lies in complementing each other’s advantages and forming a collaborative intervention model from macro to micro. The core advantage of modern medical treatment is to rapidly improve hemodynamics and reduce cardiac load, which provides the necessary basic conditions for the restoration of intestinal perfusion and the improvement of the microenvironment. However, modern medicine struggles to directly repair the damaged intestinal barrier or correct deep dysbiosis. On this basis, TCM gives full play to the advantages of multi-component and multi-target, and blocks the continuous damage of enterogenic inflammation and metabolic toxicity to the myocardium from the source by providing prebiotic substrates, repairing the intestinal barrier, and regulating microbiota metabolism. The specific research shows the following:

Modern medical conventional treatment combined with Yiqi Fupai formula significantly improved cardiac function and delayed ventricular remodeling in HF mice through dual regulation of the gut-heart axis (Zhang Z. X. et al., 2025). Mechanistically, in a murine HF model, Yiqi Fupai formula was found to enrich the abundance of beneficial bacteria such as *Akkermansia muciniphila* and *Bifidobacterium* in the gut, promote SCFAs synthesis, and simultaneously upregulate ZO-1 and Occludin expression, reducing gut microbiota translocation (Zhang Z. X. et al., 2025). Meanwhile, *β*-blockers improve intestinal perfusion, providing a favorable microenvironment for the remodeling of gut microbiota. The two work synergistically to regulate gut microbiota structure by improving hemodynamics, repairing the intestinal barrier, and ultimately delaying the progression of HF.

The combined use of ACEI and Danshen injection, through network meta-analysis and basic research, has been shown to reduce myocardial collagen deposition and lower serum TMAO levels more effectively than ACEI alone (Song et al., 2024; Liu et al., 2022). ACEIs reduce Ang II-mediated intestinal mucosal damage by inhibiting the RAAS system, indirectly improving the microbial colonization environment; Danshen-based TCMs directly downregulate the abundance of TMAO-producing bacteria such as *Clostridium difficile* in the intestine, reducing the toxic effects of TMAO on the myocardium (Shao et al., 2022). This dual approach inhibit the activation of the TLR4/NF-κB pathway, thereby reducing myocardial inflammation and fibrosis.

In HF patients, the combination of modern medical basic treatment and Shenfu Qiangxin Wan (a TCM formula) significantly improved symptoms such as cold intolerance and lower extremity edema, while increasing LVEF, reducing serum CRP and TNF-*α* levels, and upregulating the expression of intestinal barrier tight junction protein (Guo et al., 2020). Although this study did not directly detect the microbial community, Shenfu Qiangxin Wan can regulate intestinal pH and osmotic pressure, inhibiting the translocation of opportunistic pathogens, while diuretics and positive inotropic agents rapidly relieve edema and reduce the damage to the microbial microenvironment caused by intestinal congestion (Guo et al., 2020; Wu J. et al., 2025), the two work together to improve the integrity of the intestinal barrier and indirectly correct the microbial imbalance.

The combined treatment of Luqi formula and modern medicine can improve HIF-1α-mediated intestinal barrier integrity, reduce endotoxin entry into the bloodstream, regulate intestinal flora composition, promote macrophage polarization from M2 to M1, and synergistically alleviate ventricular remodeling in the stress overload-induced HF model (Wang X. et al., 2025; Yan et al., 2023; Guo J. et al., 2025). In this combination, ARBs alleviate intestinal microcirculatory disorders, while Luqi Fang enriches SCFA-producing bacteria through polysaccharide components, and SCFAs further activate intestinal GPR43 receptors, inhibiting the release of inflammatory factors (Guo J. et al., 2025).

A systematic review and meta-analysis of 28 RCTs evaluating ginseng-containing TCM for acute decompensated HF reported that combined treatment significantly improved clinical efficacy and LVEF compared to conventional Western therapy alone (Chen et al., 2022). Although the pooled analysis showed a trend toward reduced 6-month rehospitalization, this did not reach statistical significance. Mechanistic studies suggest that ginsenosides in ginseng can be metabolized into active ingredients by gut microbiota, enriching SCFA-producing bacteria such as *Lactobacillus* and *Bifidobacterium*. SCFAs exert a protective effect by inhibiting intestinal inflammation and improving myocardial energy metabolism (Chen et al., 2022), combined with the rapid and stable hemodynamics under the conventional treatment of modern medicine, it creates conditions for microbial metabolism and functional repair, and the two work together to improve long-term prognosis.

In summary, the synergistic effect of integrated traditional Chinese and Western medicine in treating HF targets both hemodynamic abnormalities and gut microbiota imbalance. Modern medicine focuses on the former for rapid symptom control, while TCM focuses on the latter for long-term, fundamental treatment. The gut microbiota, as a key link in this process, mediates intestinal barrier repair, inflammation suppression, and metabolic optimization, amplifying the hemodynamic efficacy of modern medicine and strengthening the overall regulatory advantages of TCM. Although the direct evidence for integrated traditional Chinese and Western medicine in regulating gut microbiota to treat HF is still fragmented and mostly based on small samples or basic research, the feasibility and potential of this approach have been clearly demonstrated.

#### 5.2.2 Detoxification effect

Modern medical anti-HF drugs and potentially cardiotoxic drugs often have serious toxic side effects while playing a therapeutic role. The mechanism of these side effects is not only related to direct cell damage, but also closely related to the drug disrupting the intestinal microecological balance and inducing a vicious circle of gut-mandribble (Branchereau et al., 2019). Integrated traditional Chinese and Western medicine treatment, by targeting the gut microbiota and repairing the intestinal barrier, can effectively block toxicity transmission pathways and improve patients’ tolerance to modern medical treatments.

To address chemotherapy-induced cardiotoxicity, TCM exerts a detoxifying effect by remodeling the gut microbiota and blocking ferroptosis. Doxorubicin (DOX) is one of the main causes of drug-induced HF, and its pathogenic mechanism involves the dual effects of oxidative stress and gut microbiota dysbiosis. Huang et al. reviewed that DOX can cause gut microbiota imbalance, leading to systemic inflammation and exacerbating myocardial damage (Huang et al., 2024). Based on this, Hu et al.’s research confirmed that the combined use of emodin and DOX can specifically remodel the gut microbiota structure. This optimization of the microbiota directly inhibited ferroptosis signaling in cardiomyocytes. If the microbiota was cleared by antibiotics, this protective effect immediately disappeared, confirming that the gut microbiota is the core target for DOX detoxification (Hu et al., 2024a). Furthermore, synbiotic supplementation has also been shown to reduce DOX-induced oxidative stress and inflammatory responses through the gut-heart axis (Cheng et al., 2025). The TCM compound Qi Shen granules reduce oxidative damage by protecting mitochondrial function and regulating the Sirtuin3 pathway (Zhang J. et al., 2024). This multi-target intervention model complements the regulation of the intestinal metabolic environment, jointly alleviating the cardiotoxicity of chemotherapy drugs.

TCM can improve the safety of cardiac glycosides (such as digoxin) by intervening in their metabolic processes, thus reducing their narrow therapeutic window and the risk of toxicity. Digoxin is a classic drug for treating atrial fibrillation with HF, but it is prone to accumulation and toxicity. A recent study by Zhuang et al. indicates that the interaction mechanism between TCM and digoxin is complex, with the metabolic transformation of the gut microbiota being a key link (Zhuang W. et al., 2023). Certain components of TCM can regulate the abundance of gut microbiota that metabolizes digoxin, or regulate drug transport by affecting the expression of P-glycoprotein (P-gp), thereby maintaining the cardiotonic effect while reducing abnormal accumulation and potential toxicity of the drug in the body. This therapeutic strategy based on regulating gut microbiota provides a new approach for the safe clinical use of digitalis drugs.

Furthermore, for endotoxemia and metabolic disorders caused by conventional treatments, TCM can exert a protective effect through barrier repair and anti-inflammation. HF itself and the use of diuretics often lead to intestinal wall edema and impaired barrier function. Studies by Zhang C. et al. (2025) have revealed that gut microbiota dysbiosis can exacerbate the inflammatory response in HF by activating the LPS-TLR4/NF-κB signaling axis. Regarding this mechanism, Lu Yanmeng et al. proposed that the gut microbiota is an important entry point for enhancing the efficacy and reducing the toxicity of TCM in clinical practice (Yan et al., 2019). For example, the classic formula Linggui Zhugan Decoction has been shown to significantly regulate gut microbiota and its metabolites, and improve lipid metabolism and inflammatory states (Zhu et al., 2023). Although the study is based on a non-alcoholic fatty liver model, its mechanism of improving edema by remodeling the flora is similar to that of HF treatment, which helps alleviate electrolyte disturbances and metabolic burden that may be caused by Western diuretic therapy, repair the damaged intestinal barrier, and thus block the re-damage to the heart caused by enterogenic inflammation. In summary, the toxicity reduction strategy of integrated traditional Chinese and Western medicine is not simply against each other, but by reshaping the gut microecology and repairing the biological barrier damage caused by drugs or diseases, so as to create a safer internal environment for modern medical treatment.

## 6 Conclusion

This study reviewed the driving role of the gut-heart axis in the pathogenesis of HF, clarifying that gut microbiota dysregulation is not only a concomitant phenomenon of HF, but also a key factor accelerating myocardial remodeling and cardiac function decline. In the whole course of HF management, gut microbiota, as the core hub of integrated traditional Chinese and modern medicine treatment, has shown significant potential for synergistic effects and toxicity reduction. We reviewed the mechanisms and evidence of how TCM and Western medicine regulate gut microbiota individually and in combination, and constructed a correlation system between Western medicine subtypes/gradings and gut microbiota characteristics.

The gut microbiota serves as a crucial hub in the integrated treatment of HFusing both traditional Chinese and Western medicine. Its synergistic mechanism achieves complementary advantages in both macroscopic microenvironment repair and microscopic microecological remodeling. Modern medicine rapidly improves cardiac load and intestinal perfusion, creating a favorable macroscopic microenvironment for the restoration and reconstruction of gut microbiota homeostasis. On the other hand, TCM blocks the transmission of gut-derived injury signals through pathways such as regulating SCFAs synthesis, inhibiting LPS translocation, and reducing TMAO toxicity. This approach effectively addresses the intestinal barrier repair and metabolic imbalance processes that are difficult for Western medicine alone to address, while simultaneously reducing drug cardiotoxicity and improving treatment safety and tolerability. It should be noted that the majority of mechanistic evidence reviewed herein is derived from preclinical studies conducted in animal models or *in vitro* systems. Large-scale, well-designed randomized controlled trials with standardized microbiota profiling are needed to validate these findings in clinical HF populations and to establish causal relationships between gut microbiota modulation and cardiac outcomes.

Despite the immense potential shown by gut-heart axis research, the following challenges remain in achieving clinical translation: 1. Metagenomics, metabolomics, and single-cell sequencing (scSeq) technologies should be integrated to identify specific microbial biomarkers reflecting the TCM treatment category, Western medicine subtype, and NYHA classification of HF, and to construct predictive gut microbiota models with clinical diagnostic value. 2. More advanced experimental models, such as gut/heart organoid co-culture systems and humanized microbiota experimental models, should be utilized to deeply analyze the interaction pathways between TCM components and specific microbial enzymes, shifting from correlation studies to deterministic causal mechanism studies. 3. Large-scale, prospective, randomized controlled clinical trials based on microbiota biomarker stratification should be conducted to verify the long-term prognostic impact of modern medical basic treatments combined with TCM-regulated microbiota regimens on patients with different metabolic backgrounds, and to accelerate the development of gut-targeted TCM preparations and specific response biopharmaceuticals.

Of particular interest, the field of medicinal food homology offers a complementary perspective for gut-heart axis intervention. Recent preclinical studies have demonstrated that linoleic acid and *α*-linolenic acid derived from floating wheat (*Triticum aestivum* L.), a traditional medicinal food, may attenuate chronic HF through AQP1 modulation and gut microbiota remodeling in murine models (Li H. et al., 2025a; Li H. et al., 2025b). These findings suggest that dietary bioactive compounds with both nutritional and pharmacological properties hold potential for reshaping the intestinal microecology and protecting cardiac function. Moreover, a clinical study has shown that a high-fiber dietary intervention guided by gut microbiota profiling can improve cardiac function and reduce systemic inflammation in patients with chronic HF (Li L. et al., 2025a). Future research should further explore the role of medicinal food homologous substances in the gut-heart axis, which may expand the repertoire of nutritional and microbiota-targeted strategies for HF management.

In summary, the gut microbiota offers a novel breakthrough for the prevention and treatment of HF, and the integrated traditional Chinese and Western medicine treatment strategy based on the gut-heart axis combines the advantages of systemic regulation and targeted intervention. With the deepening of mechanistic research and the accumulation of clinical evidence, this treatment strategy is expected to become an important component of comprehensive HF management, providing a practical new path to improve patient prognosis and reduce the disease burden.

## Glossary

ACEI: Angiotensin-Converting Enzyme Inhibitor
AMPK: Adenosine Monophosphate-Activated Protein Kinase
ARB: Angiotensin Receptor Blocker
CHF: Chronic Heart Failure
DMB: 3,3-Dimethyl-1-butanol
DOX: Doxorubicin
eNOS: Endothelial Nitric Oxide Synthase
F/B Ratio: Firmicutes/Bacteroidetes Ratio
FMO3: Flavin-Containing Monooxygenase 3
FXR: Farnesoid X Receptor
H₂S: Hydrogen sulfide
HF: Heart Failure
HFpEF: Heart Failure with preserved Ejection Fraction
HFrEF: Heart Failure with reduced Ejection Fraction
IL: Interleukin (e.g., lL-1*β*, 1 L-6, 1 L-10)
ImP: Imidazole Propionate
IPA: Indole-3-Propionic Acid
ISO: Isoproterenol
LPS: Lipopolysaccharide
LVEF: Left Ventricular Ejection Fraction
mTORC1: Mammalian Target of Rapamycin Complex 1
NF-κB: Nuclear Factor-κB
NLRP3: NOD-Like Receptor Protein 3
NOX: NADPH oxidase
NYHA: New York Heart Association
PAA: Phenylacetic acid
PAGln: Phenylacetylglutamine
ROS: Reactive Oxygen Species
SCFAS: Short-Chain Fatty Acids
SID: Selective Intestinal Decontamination
TCM: Traditional Chinese Medicine
TGF-β: Transforming Growth Factor-β
TGR5: Takeda G protein-coupled receptor 5
TLR4: Toll-Like Receptor 4
TMAO: Trimethylamine N-Oxide
TNF-α: Tumor Necrosis Factor-α
Treg: Regulatory T Cell
ZO-1: Zonula Occludens-1
